# The impact of conflict on infectious disease: a systematic literature review

**DOI:** 10.1186/s13031-023-00568-z

**Published:** 2024-04-08

**Authors:** Valia Marou, Constantine I. Vardavas, Katerina Aslanoglou, Katerina Nikitara, Zinovia Plyta, Jo Leonardi-Bee, Kirsty Atkins, Orla Condell, Favelle Lamb, Jonathan E. Suk

**Affiliations:** 1 School of Medicine, University of Crete, Heraklion, Crete Greece; 2 Department of Oral Health Policy and Epidemiology, Harvard School of Dental Medicine, Harvard University, Boston, MA USA; 3 Centre for Evidence Based Healthcare, School of Medicine, University of Nottingham, Nottingham, UK; 4 Emergency Preparedness and Response Support, European Centre for Disease Prevention and Control, Solna, Sweden

**Keywords:** Conflict, War, Infectious diseases, Outbreaks, Control, Prevention, Preparedness, Strategies

## Abstract

**Background:**

Conflict situations, armed or not, have been associated with emergence and transmission of infectious diseases. This review aims to identify the pathways through which infectious diseases emerge within conflict situations and to outline appropriate infectious disease preparedness and response strategies.

**Methods:**

A systematic review was performed representing published evidence from January 2000 to October 2023. Ovid Medline and Embase were utilised to obtain literature on infectious diseases in any conflict settings. The systematic review adhered to PRISMA (Preferred Reporting Items for Systematic Reviews and Meta-analysis). No geographical restrictions were imposed.

**Findings:**

Our review identified 51 studies covering AIDS, Hepatitis B, Tuberculosis, Cholera, Coronavirus 2, Ebola, Poliomyelitis, Malaria, Leishmaniasis, Measles, Diphtheria, Dengue and Acute Bacterial Meningitis within conflict settings in Europe, Middle East, Asia, and Africa since October 2023. Key factors contributing to disease emergence and transmission in conflict situations included population displacement, destruction of vital infrastructure, reduction in functioning healthcare systems and healthcare personnel, disruption of disease control programmes (including reduced surveillance, diagnostic delays, and interrupted vaccinations), reduced access by healthcare providers to populations within areas of active conflict, increased population vulnerability due to limited access to healthcare services, and disruptions in the supply chain of safe water, food, and medication. To mitigate these infectious disease risks reported preparedness and response strategies included both disease-specific intervention strategies as well as broader concepts such as the education of conflict-affected populations through infectious disease awareness programmes, investing in and enabling health care in locations with displaced populations, intensifying immunisation campaigns, and ensuring political commitment and intersectoral collaborations between governments and international organisations.

**Conclusion:**

Conflict plays a direct and indirect role in the transmission and propagation of infectious diseases. The findings from this review can assist decision-makers in the development of evidence-based preparedness and response strategies for the timely and effective containment of infectious disease outbreaks in conflict zones and amongst conflict-driven displaced populations.

**Funding:**

European Centre for Disease Prevention and Control under specific contract No. 22 ECD.13,154 within Framework contract ECDC/2019/001 Lot 1B.

**Supplementary Information:**

The online version contains supplementary material available at 10.1186/s13031-023-00568-z.

## Introduction

Military conflicts characterised by war have had a significant impact on healthcare infrastructure and systems [1, 2]. Affected populations may be subjected to periodic outbreaks of violence (lasting weeks to months), ongoing or recurring insecurity in a protracted conflict (lasting years to decades), or long-term ramifications of previous (usually prolonged) war [1].

In addition, populations in conflict situations present increased incidence of infectious diseases as a result of a multitude of risk factors that precipitate disease emergence and transmission [2]. These conflict-related factors include the disruption of vital and health infrastructures and large-scale, forced population movements that further challenge resources in affected countries and aid disease emergence and transmission [1, 3]. Infectious disease outbreaks in conflict settings present a unique challenge to public health and emergency response. Detection and control of many emerging infectious diseases in conflict situations require a functional healthcare system with a sufficient number of trained healthcare workers and adequate supplies of medications, vaccines, and equipment [1, 4]. Thus, delays in the detection, response, and containment of an infectious disease outbreak in countries affected by conflict prolong the suffering of the population of the country and elevate the risk of the transmission of infectious diseases to surrounding countries and to countries globally [4].

The Conflict in Ukraine, which started in early 2022 during the COVID-19 pandemic, reminded the world of the risks associated with infectious disease outbreaks among displaced populations and emphasised the significance of having an emergency preparedness plan and response system in place to address infectious disease outbreaks in conflict regions [5]. Considering the former, this systematic literature review examines the pathways through which infectious diseases emerge in conflict situations and assesses preparedness and response strategies with the aim of informing the work of public health agencies and countries affected by protracted conflicts.

## Methods

The systematic review adhered to PRISMA (Preferred Reporting Items for Systematic Reviews and Meta-Analysis) presented in Supplementary Table 1 [6]. The protocol of this systematic review was pre-reviewed by the European Centre for Disease Prevention and Control. The protocol was not pre-registered in any database for systematic reviews.

### Outcomes and inclusion/exclusion criteria

Studies of all study designs, including field reports and perspective articles, with no geographical limitation were considered eligible provided they evaluated infectious diseases in conflict-affected countries and were published in English between January 2000 and October 2023 (Supplementary Table 2).

### Study selection

Relevant studies were identified within Ovid Medline and Embase. Subject heading terms and free text words were used to develop a comprehensive search strategy which is presented in Supplementary Table 3. Studies that met the search criteria were evaluated for their validity and reliability. Systematic and non-systematic literature reviews were excluded, but their references were screened. Initially, a pilot round of title/abstract screening was conducted, where a random sample of 100 titles was screened for eligibility independently by two reviewers (ZP, KA) to enable consistency in screening and to identify areas for amendments in the inclusion criteria. A high measure of inter-rater agreement was achieved (percentage agreement > 90%), hence the remaining titles were distributed to be screened independently by two reviewers. For the full-text screening, all full texts were screened for eligibility independently by two reviewers (KN, KA). Any disagreements were discussed with a third reviewer (CV). Documents that passed the inclusion criteria on the full-text screening were included in the review.

### Data extraction, synthesis, and presentation

Data were extracted independently by two reviewers (VM, CV) using a predesigned data extraction sheet. Any discrepancies were discussed and agreed upon. The extracted data were organised in a tabular format and included: study characteristics (first author’s name, year of publication), geographical context (country/area), setting, population characteristics, sample size, methodology/study type, and numerical/ descriptive findings regarding type of infectious disease, “conflict-to-infectious disease pathways”, and measures implemented and/or suggested to mitigate outbreaks. A qualitative analysis of the included literature was performed. To evaluate the data and describe each study, a narrative synthesis approach organised by infectious disease category was utilised.

### Assessment of study quality

The methodological quality of the included studies was evaluated independently by two reviewers (VM, KA) using the appropriate Joanna Briggs Institute (JBI) standardised critical appraisal tools [7]. Any points of uncertainty were addressed through discussion and consensus with a third reviewer (CV). The results of the quality appraisal are presented in Supplementary Table 4.

## Results

**Fig. 1:**
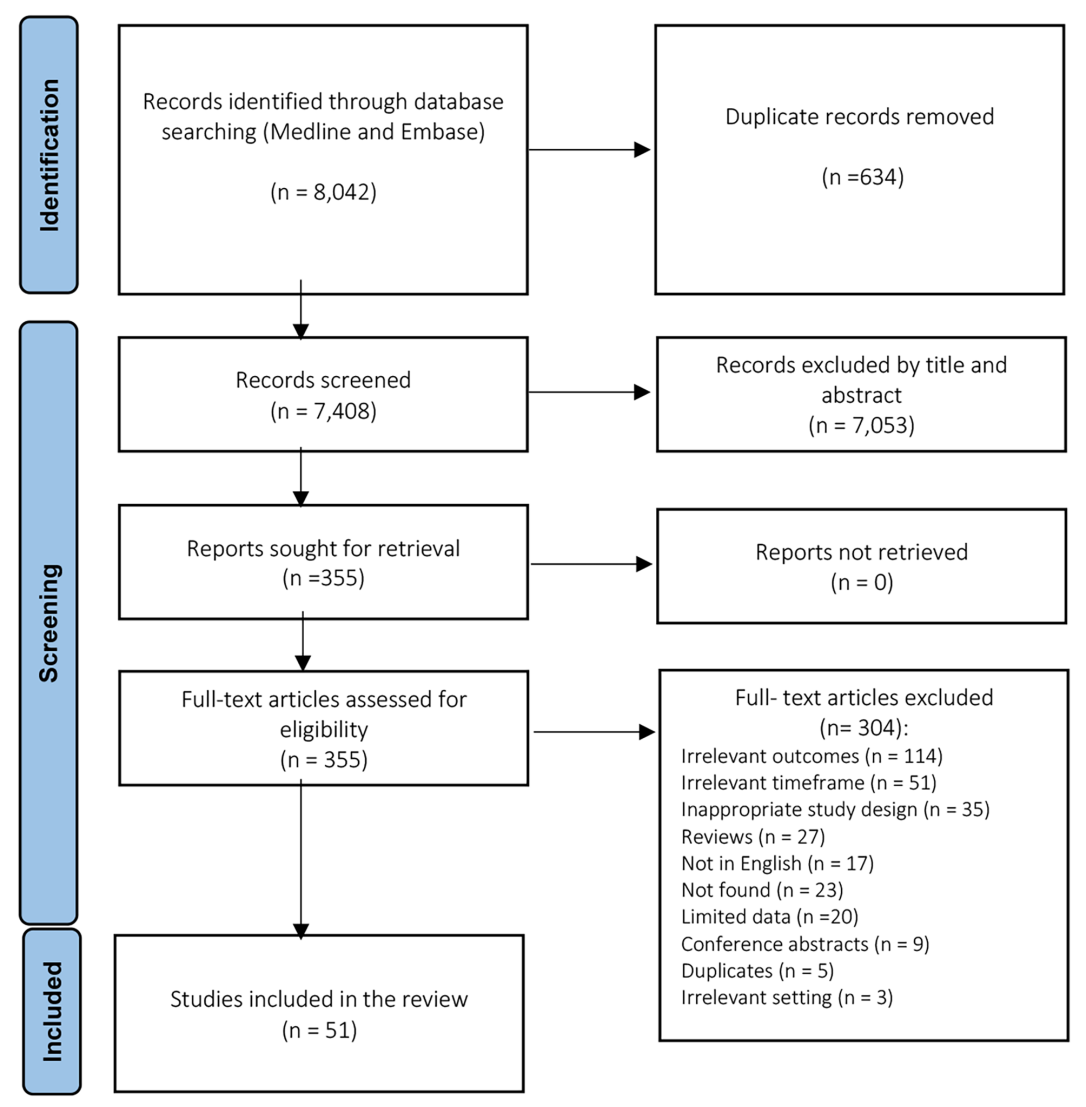
Flowchart of study selection for the current review

A total number of 8,042 studies were identified according to the specified selection criteria in Ovid MEDLINE and Embase. After removing duplicates, 7,408 were screened by title and abstract, out of which 355 studies were assessed for full-text eligibility. Through the assessment of the full texts, 304 studies were excluded due to limited data, language and timeframe restrictions, irrelevant outcomes, settings, and study types (reviews, conference abstracts). Consequently, 51 studies were eligible to be included in this current systematic review as depicted in the PRISMA flowchart in Fig. 1.

### HIV and HBV

Our systematic review identified five studies reported in Table 1, on Human Immunodeficiency Virus (HIV)/ Hepatitis B virus (HBV) in association with conflict published within the timeframe January 2000– October 2023 [9–13]. A higher incidence rate of HIV was reported in the conflict-torn regions of Cote d’Ivoire [9], Libya [10], Ukraine [11], and Uganda [12], as the result of population displacement [9–11], continuous interruption of healthcare services [10], overall decrease in healthcare personnel (especially in medical doctors), and reduction in functioning health facilities [9]. Additional effects of the armed conflict escalating HIV prevalence in Cote d’Ivoire had been the plundered healthcare delivery structures and the disappearance of laboratory equipment and surveillance data [9]. Conflict-affected participants who had experienced abduction and multiple traumas during the war were reported to be at a greater risk of HIV infection [12]. It has been noted that human resource and financial constraints, lack of equipment, diagnostic kits for sexually transmitted infections, essential drugs, reduction in the number of condoms sold and the lack of awareness campaigns were major factors impeding the implementation of effective HIV/ Acquired Immunodeficiency Syndrome prevention activities in war-torn areas [9]. In Pakistan, the prolonged armed conflict was reported to have caused an increase in poverty, medical deprivation, uncertainty, and a breakdown of social structures that facilitated the transmission of HBV [13]. High prevalence of HBV was observed in areas with high frequency of military activities [13]. Finally, the low socioeconomic status and the lack of basic health facilities were risk factors associated with HBV infection [13].

**Table 1:** Impact of conflict on HIV and HBV outbreaks

*Author*	*Country, Setting, Timeframe*	*Population*	*Conflict to Disease Pathways*	*Prevention and Preparedness Strategies Suggested/Implemented*
Betsi et al., 2006 [9]	Armed conflict in Cote d’Ivoire (Central, North and West areas), 2001- early 2004	Key Informant survey (*n* = 165) among politicians, military leaders, health staff, members of organisations to address HIV, NGOs	• Population displacement (25-50%, depending on the part of the country) • Decrease in healthcare personnel (75–88% reduction), especially medical doctors (91–98%) • Reduction in functioning health facilities (72–80%) • Healthcare delivery structures had been plundered or destroyed, laboratory equipment had been stolen and patient records and epidemiological monitoring and surveillance data had disappeared • Lack of essential drugs and diagnostic kits for STIs • Low compliance to diagnostic algorithms • Lack of patient visitation • Lack of awareness campaigns • Interrupted condom distribution • Interruption of existing antiretroviral therapy programmes	***Implemented***: • An Increased number of active NGOs pursued education and sensitising programmes for the prevention and care of people living with HIV/AIDS. ***Suggested***: • Awareness campaigns with emphasis on the age group 15–24 were suggested. • Health facilities to be structurally and functionally rehabilitated to provide people living with HIV/AIDS antiretroviral treatment and other STIs in the long term.
Ali et al., 2012 [13]	Pakistan, conflict affected area in North Waziristan Pakistan, 2010–2011	Population of the conflict-affected area	• The prolonged armed conflicts caused a reported increase in poverty, medical deprivation, uncertainty and the breakdown of social structures that facilitated the transmission of HBV. • Low socioeconomic status, and illiteracy were associated with HBV. • High HBV prevalence was observed in areas with high frequency of military activities • Transmission factors within this setting were the reuse of needles and syringes, sexual exposure, barbers’ shops, tattooing	***Implemented***: Not mentioned ***Suggested***: • Vaccination and awareness programmes are necessary to prevent the HBV epidemic
Vasylyeva et al., 2018 [11]	Ukraine, Earlier stages of the Ukraine conflict, 24 regional AIDS centres, 2012–2015	Patients from Ukrainian AIDS centres	• The conflict internally displaced HIV-infected people • In conflict areas, healthcare provision and harm-reduction services were interrupted • The stress of the displacement might result in treatment failures for HIV-infected patients • Patients who had to relocate because of the conflict may be more likely to reduce treatment adherence or drop out of treatment for some time • Virus dissemination due to population movement was directed to the locations with the highest prevalence of people who inject drugs practising risky sexual behaviours	***Implemented***: Not mentioned ***Suggested***: • Enabling sustainable prevention services and treatment provision in locations where services have been physically disrupted. • Proactive and routine integration of HIV testing for people who have relocated due to the war, or who frequently travel to the war zone. • Scale-up of harm reduction services for people who inject drugs will be an important factor in preventing new local HIV outbreaks in Ukraine.
Katamba et al., 2020 [12]	Uganda, Post-conflict Northern Uganda, November 2011 - March 2015	Conflict-affected population in three districts in Northern Uganda	• Conflict-affected participants who had experienced abduction and multiple traumas during the war were at greater risk of HIV infection.	***Implemented***: Not mentioned ***Suggested***: • Trauma-informed HIV prevention and treatment services, and culturally safe mental health initiatives are needed
Daw et al., 2022 [10]	Libya, Libyan armed conflict, 2011–2020	People from four regions of Libya (East, West, North, and South)	• Healthcare services were continuously interrupted • Internal population displacement (25%) leading to geographic spread of HIV virus from the regions involved in the armed conflict to the rest of the country. • Population displacement may be reflected in the reduced treatment of HIV-infected individuals as patients who had to relocate because of the conflict may be more likely to reduce treatment adherence.	***Implemented***: Not mentioned ***Suggested***: • National intervention policies during and at post-conflict periods should be implemented • Geographically tracing interventions should be introduced • Viral treatment therapy to those infected should be introduced all over the country • A national registry system for all infected patients to support access to care

Abbreviations: AIDS = Acquired Immunodeficiency Syndrome, HBV = Hepatitis B, HIV = Human Immunodeficiency Virus, NGOs = Non- Governmental Organisations, STIs = diagnostic kits for sexually transmitted infections

With the exception of the Non-Governmental Organisations’ (NGOs) active involvement in the prevention of and care for people with HIV in Cote d’Ivoire [9], there was no reported implementation of infection prevention protocols in the included literature. However, preventive measures were suggested and included vaccination [13], awareness campaigns [9, 13] with emphasis on age group 15–24 year olds [9], and rehabilitation of health facilities to provide people living with HIV/AIDS antiretroviral treatment and STIs in the long term [9]. Furthermore, proactive HIV testing was suggested for internally displaced people (IDPs) and people who frequently travel to war-affected areas to be included in effective preventative measures [11]. Also, harm reduction services were suggested as significant preventative measures for HIV outbreaks among people who inject drugs. Finally, the importance of enabling sustainable prevention services and treatment provision in locations where services have been physically disrupted because of the armed conflict was also stressed [11].

### Cholera

The current systematic review identified eleven studies, outlined in Table 2, on cholera and conflict published between January 2000 and October 2023 [14–24]. In the conflict-affected regions of Monrovia, Liberia [17], cholera transmission was caused most likely by a severe shortage of clean water (as the piped water distribution system was deemed inactive), inadequate sanitation, and overcrowding. The above pathways were compounded by weather conditions as regional flooding washed contaminated water into shallow unprotected wells [17]. Due to the conflict in Yemen a massive internal population displacement occurred [18, 19, 24] and the population had to face insufficient shelter [16], limited access to safe drinking water, shortages of food [14, 16, 18], poor sanitation, destruction of healthcare facilities [14, 16, 19, 24], disruption in sewage management and wastewater treatment facilities, and a lack of electricity to power water pumps [16, 18]. Compounding environmental factors (rainfall, flooding, and water contamination) were noted in Yemen as well [14]. In Iraq, the armed civil war dispersed a large number of IDPs which, combined with the influx of Syrian refugees into the country (a result of the Syrian civil war), ultimately led to overcrowded shelter arrangements and limited access to drinking water, safe food, and basic healthcare services [15]. These factors greatly contributed to the cholera transmission in the region [15].

**Table 2:** Impact of conflict on Cholera outbreaks

*Author*	*Country, Setting, Timeframe*	*Population*	*Conflict to Disease Pathways*	*Prevention and Preparedness Strategies Suggested/Implemented*
Center for Disease Control and Prevention (CDC) 2003 [17]	Monrovia, Liberia, June 2003 –September 2003	Population of Monrovia, Liberia	• Acute shortage of clean water as the piped water distribution system was deemed inactive • Poor sanitation • Crowded living conditions • Weather conditions as regional flooding washed contamination into shallow, unprotected wells	***Implemented***: International and Liberian organisations attempted to supply IDP settlements with sufficient potable water and began chlorinating wells ***Suggested***: • Provision of increased amounts of clean water • Health education • Chlorination of water in protected household containers
Altmann et al., 2017 [24]	Hodeidah city, Yemen, Al Thowra hospital, 28 October 2016–28 February 2017	Population of Hodeidah city, Yemen	• 2 million IDPs • 462,000 children with Severe Acute Malnutrition • Half of its population without access to safe drinking water and • 14.8 million with no access to health care services (only 45% of health facilities are functional).	***Implemented***: NGO Action Contre la Faim with Yemen’s Ministry of Public Health and Population responded to the epidemic: • Provided physical space and key staff, • The construction and/or rehabilitation of health centres, • Staff recruitment (nurses, cleaners, pharmacists, logisticians, WASH workers), • Supervision and training, • Supply chains for drugs and medical materials, • Set up support systems (logistics, WASH, data entry and analysis), • Human Resource management, financial resources to roll out the intervention and clinical supervision • Access to safe water through water trucking • Provision of hygiene education including hand washing and waste disposal at water points ***Suggested***: Not mentioned
Lam et al., 2017 [15]	Iraq, Refugee camps, 2015	People from 27 refugee camps in 10 governorates	• Large numbers of IDPs residing in camps, informal settlements, or temporary placement sites (collective centres) • Influx of Syrian refugees • Overcrowded, inadequate shelter arrangements and limited access to sanitation facilities, safe drinking water, safe food, and basic healthcare services	***Implemented***: • Implemented WASH and other cholera control measures • Oral cholera vaccines uptake in IDP camps at full capacity or overcrowded and to all refugee camps and collective centres • The use of the global OCV stockpile intended to provide rapid deployment of OCVs in emergency and outbreak situations managed by an International Coordination Group
Qadri et al., 2017 [16]	Yemen, December 2016 – September 2017	Yemenis	• Inadequate shelter • Inadequate sanitation • Shortages of water • Shortages of food • Shortages of medical supplies • Shortages of fuel • Destructed healthcare facilities • Disruption of sewage management and wastewater treatment facilities • Lack of electricity to run water pumps	***Implemented***: Cooperation between WHO, UNICEF, other international agencies, nongovernmental organisations, and Yemeni healthcare providers to restore the operationalisation of water-treatment plants, provide hygiene kits with soap and chlorination tablets, and provide training in water-sanitation–hygiene behaviours to help prevent cholera ***Suggested***: • To create and deploy the OCV global stockpile • Develop predictive tools to identify humanitarian emergencies posing a high risk of cholera
Al-Mekhlafi, 2018 [18]	Yemen, Civil war in Yemen, 2 October 2016–14 January 2018	Population of Yemen	• 7.3 million severely food insecure • 3.3 million IDPs • 55% of health facilities partially functioning or destroyed • Airport closures • Severe shortages of fuel, food, drinking water, and medication • Existing shortage of water before the conflict • Clogged sewage and drainage systems • Waste disposed of in the streets • Underground water in all Yemeni cities is contaminated with sewage and treatment plants are not functioning because of lack of fuel and maintenance	***Implemented***: Yemen government, United Nations, and WHO stated that they should be focused on a WASH intervention to provide safe water and sanitation, setting up diarrhoea treatment centres, and improving the population’s awareness about the disease. ***Suggested***: • An intense vaccination strategy and provision of stockpiled vaccines was suggested • Continuation of the WASH programme • Timely establishment of diarrhoea treatment centres and oral rehydration points • Provision of therapeutic and diagnostic supplies and fuel to health facilities • Community mobilisation through awareness campaigns • Assessment of strains and dynamics to evaluate spatial and temporal transmission (monitoring)
Dureab et al., 2018 [19]	Yemen, 17 directorates, 2016 (Week 39–52, 2016)	Population of Yemen (*n* = 15,074 cholera cases)	• Conflict related factors (destruction, casualties), • IDPs (outgoing and returning) • Water and sanitation disruption • Poor infrastructure	***Implemented***: Not mentioned ***Suggested***: • Distributing public awareness materials on proper personal hygiene, food and water safety • Improving the preparedness of the public health authorities for surveillance (including public health laboratories at central and regional levels) and response systems • Arrangements for leadership and coordination • Preparedness of case definitions, rapid testing kits, case management procedures, • Stockpiles of medical supplies • Establishment of a community surveillance system with an awareness and prevention component would aid in spotting the early indicators of morbidity and mortality and slow the spread of cholera, especially in the context of Yemen.
Jones et al., 2020 [20]	South Sudan, June 2014 – December 2017	People in South Sudan	• Large-scale population movements between counties of South Sudan with cholera outbreaks • Movement from neighbouring countries • Large-scale population displacement and movement partially explained the differences in the number of cases between years • Synergistic effects with precipitation and climatic determinants • Cholera control efforts during these outbreaks was continually hampered by conflicts and restricted access to areas with ongoing transmission	***Implemented***: • Case management, surveillance, • WASH interventions, hygiene promotion, and enforcement of sanitation standards • Chlorination of public water sources, in public areas • OCV was administered in South Sudan through 36 vaccine campaigns • Phylogenetic analyses to trace the geographical spread of infection ***Suggested***: • Regional-level responses to curb outbreaks of cholera in humanitarian settings. • OCV campaigns • Interventions to improve water, sanitation, and hygiene in vulnerable settings, • Controlling cholera in nearby countries that have the potential to introduce cholera might be an effective additional strategy • Increased whole genome sequencing to support surveillance and understanding the spread of infections • Improved methods for measuring population movement within and between countries during complex emergencies is needed
Simpson et al., 2022 [14]	Yemen, Yemen and 20 Yemeni governorates, 4 September 2016–29 December 2019	Yemenis	• Limited access to health care and damaged health infrastructure depleted medical resource stockpiles • Limited access to safe and affordable water • Growing malnourished and immunocompromised population increased the risk of infection • Compounding environmental factors with the underlining conflict related damage (i.e. rainfall, flooding and water contamination). • Reduced availability of resources due to other epidemics (i.e. SARS-CoV-2)	***Implemented***: Not mentioned ***Suggested***: • Utility of surveillance data to characterise, classify, and compare infectious disease outbreak signatures to examine spatiotemporal patterns and perform a vulnerability mapping of outbreak hotspots to improve resource management and mobilisation during humanitarian aid responses. • Public sharing of epidemiological information
Ahmed et al., 2022 [21]	Syrian Arab Republic	Syrian population	• Numerous laboratory facilities, healthcare units, water plants, and sewerage systems were compromised due to airstrikes, and millions left displaced and forced to reside in overcrowded, poorly hygienic refugee camps • Increasing water scarcity, due to drought and reduced groundwater, and escalating dependence on unsafe water due either to armed encroachment of power supplies to central water stations or to the dependence on unmonitored resources such as private vendor trucks	***Implemented***: Not mentioned ***Suggested***: • Government and opposition groups must be convinced to ease passages of assistance • Importance of cumulative efforts to improve safe water access, sewerage systems, healthcare facilities, nationwide surveillance system, and infrastructure. • In times of instability and conflict, the expansion of the water supply by private trucks appears to be the only feasible option to meet the population’s demands. • The local population must be encouraged to ensure optimal hygiene by boiling and chlorinating the water, if available.
Al-Tammemi & Sallam 2023 [22]	Syria September 2022- November 2022	Syrian population	• War and its violence collapsed infrastructure, affecting water and sanitation infrastructure forcing people to rely on unsafe water sources resulting in rapid spread of cholera to many governorates	***Implemented***: Not mentioned ***Suggested***: • A WASH response must be implemented with inter-agency and multisectoral coordination. • Humanitarian agencies should assist by providing medical and laboratory supplies (including cholera vaccines)
Ahlaffar, et al., 2023 [23]	Syria August 2022- April 2023	Syrian population	• The armed conflict resulted in a destroyed and understaffed healthcare system with limited resources and lack of coordinated response, as well as population displacement	***Implemented***: Not mentioned ***Suggested***: • The provision of safe water and improved sanitation and hygiene practices must be urgently implemented to prevent further spread of the disease and reduce preventable deaths • improve the testing and reporting capacity of the health system and strengthen the surveillance systems to detect and respond to outbreaks promptly • Coordinated efforts and collaboration between local health authorities and international organisations working in Syria are important. • International organisations should provide technical and financial support to strengthen the country’s response, including training and equipping healthcare workers, improving disease surveillance, and expanding access to testing and treatment. • Effective community engagement is critical for the success of any disease prevention and control program, particularly in conflict-affected settings where trust in government and healthcare systems may be low • Economic development and universal access to sustainable safe drinking water and adequate sanitation, including the improvement of environmental conditions, the rehabilitation of damaged health facilities, and the improvement of early warning systems should be prioritised • The main priority must be rebuilding the country’s health system and increasing access to safe drinking water and sanitation facilities, particularly in conflict-affected areas

Abbreviations: IDPs = internally displaced people, NGO = Non-governmental organisation, OCV = oral cholera vaccine, WASH = water- sanitation- hygiene, WHO = World Health Organisation, UNISEF = United Nations International Children’s Emergency Fund, SARS-CoV-2 = Severe Acute Respiratory Syndrome Coronavirus 2

The war in Syria collapsed infrastructure including healthcare infrastructure, leaving healthcare understaffed and with limited resources [23]. This extended to water and sanitation infrastructure forcing the population to rely on unsafe water sources and unmonitored water resources (such as private vendor trucks) and resulted in the rapid spread of cholera [22]. Non-operational sewerage systems acerbated by the lack of access to proper water, sanitation, and hygiene (WASH) due to either physical destruction of established water systems or migration into insufficient and crowded camps further exposed residents to waterborne diseases, while the lack of laboratory testing facilities, healthcare personnel and surveillance further facilitate cholera outbreaks [21], aspects which were additionally impacted by the 2023 earthquake [25].

In South Sudan, the large-scale population displacement and movement (both within the country and from neighbouring countries) due to civil war partially explained the differences in the temporal and geographical cholera transmission patterns, together with the synergistic effects of precipitation and climatic determinants that aided bacterial transmission and spread [20].

Overall, response to cholera outbreaks were based on the cooperation of NGOs and governmental healthcare providers [17−18, 20] who organised access to safe water through water trucking [21, 24], water-sanitation-hygiene (WASH) interventions [15, 18, 20, 22, 24], health-hygiene education [16–18, 20, 24], chlorination of public water sources [17, 20] case management [20], surveillance through phylogenetic analyses [20], and the provision of oral cholera vaccine (OCV) [20, 24]. Additional suggestions for optimal cholera control included awareness campaigns [18], distribution of public awareness material on proper personal hygiene, food, and water safety [19], improved preparedness of the public health authorities for surveillance (including public health laboratories at central and regional levels and community surveillance systems) and response systems [19, 21, 23], preparedness of case definitions [19], rapid testing kits [19], arrangements for leadership and coordination [19, 23], and case management procedures [19]. Finally, the authors recommended economic development [23], the creation and deployment of stockpiles of medical supplies [19], the OCV global stockpile [16], the development of predictive tools to identify humanitarian emergencies [16], and utilisation of improved methods for measuring population movement within and between countries during complex emergencies [20].

### COVID-19

The current review identified six studies published between January 2000 and October 2023 related to COVID-19 in conflict settings [26–31], presented in Table 3. COVID-19 outbreaks occurred in the conflict-affected regions of Libya [26, 28], Ukraine [27, 30, 31], and Cameroon [29]. The armed civil war in Libya hindered access to populations and thus masked the actual status of the pandemic, particularly in cities devastated by the ongoing conflict where no cases of COVID-19 were reported since no health authority could work there [26]. In addition, it caused deterioration of the healthcare infrastructure, inadequate human and financial support, inadequate health facilities with limited bed capacity, lack of readiness for health emergency services, and population mobility due to displacement, all of which were reported as high risk Severe Acute Respiratory Syndrome Coronavirus 2 (SARS-CoV-2) transmission factors in Libya [28]. While lockdown measures and isolation procedures within main cities were implemented, the sharing of resources towards the conflict led to inadequate surveillance and response systems [26]. In Ukraine, the Russian invasion destroyed the healthcare infrastructure causing severe constraints such as power outages and oxygen shortages [31], damaged primary healthcare facilities [31], and led to mass migrations with people seeking refuge in confined subway systems or relocating to more secure locations [31], factors reported to have impacted SARS-CoV-2 transmission. Furthermore, the war was reported to have adversely affected Ukraine’s response to the COVID-19 pandemic through the allocation of resources to warfighting efforts [30], the reduction in medical personnel [27], reduction in hospital beds due to the need for emergency care to war wounded [27], limited testing [27], limited recording of cases in active conflict areas [27], no medicine delivery due to active hostilities [27], no application of social distancing due to high population density during the evacuation (in trains, stations, shelters) [27], limited application of personal protective measures in shelters [27], and poorly equipped health-care system in occupied territories [30]. The halting of vaccination plans in active conflict areas and the slow vaccination rollout within the other areas of Ukraine were attributed partially to the burden on medical institutions caused by the number of IDPs and the fleeing of medical staff to neighbouring countries [27]. The armed conflict in the Northwest region of Cameroon created destructive conditions that exacerbated the COVID-19 pandemic, including the internal population displacement, the destruction of health facilities, the killing of healthcare workers, the disruption of the regional healthcare system, and difficulties in delivering vaccines in security-compromised areas [29].

**Table 3:** Impact of conflict on COVID-19 outbreaks

*Author*	*Country, Setting, Timeframe*	*Population*	*Conflict to Disease Pathways*	*Prevention and Preparedness Strategies Implemented/ Suggested:*
Daw et al., 2020 [26]	Libya, 25 March 2020–25 June 2020	Population of Libya	The armed conflict: • hindered access to populations and thus masked the actual status of the pandemic, particularly in cities such as Tarhona, Tawerga and Sert, which have been devastated by the ongoing conflict and in which no official health authority could work and no cases of COVID-19 have been reported • caused population movement that spread the virus to counties located over 100 km away from the fighting such as Sebha	***Implemented***: • Lockdown measures and isolation procedures within main cities ***Suggested***: • Mapping the disease to enable the national authorities to ensure effective implementation of protective infectious disease interventions • Applying internationally accepted standards, guidelines and tools adapted to conflict situations • Specific training of health planners and health facility staff, and rapid mobilisation of international experts to provide technical field support • Effective public education programmes
Elhadi and Msherghi, 2020 [28]	Libya, Civil war in Libya, 24 March 2020–12 May 2020	Population of Libya	• Healthcare infrastructure deteriorated • Inadequate human and financial support, • Inadequate health facilities with limited bed capacity and lack of readiness of health emergency services • Population mobility due to displacement • Limited public knowledge and awareness of COVID-19	***Implemented***: Not mentioned ***Suggested***: • Encourage the support of healthcare workers by providing adequate training and personal protective equipment, • Increasing the capacity of diagnostic tools and supplies, establishing isolation sites • Increasing local awareness among the Libyan population
Chumachenko and Chumachenko, 2022 [27]	Ukraine, War in Ukraine, From 24 February 2022	Population of Ukraine	• Destruction of medical facilities and shortages of medical personnel • Reduced beds due to emergency medical care to the wounded • Limited testing and recording of cases in active conflict areas • Reduced access to oxygen and hospital beds which are prioritised for the wounded • No medicine delivery due to active hostilities • Non-application of social distancing measures due to high population density during the evacuation, both in trains and at stations and within shelters • Limited application of personal protective measures or self-isolation policies in shelters • Lack of vaccination plans in active conflict areas • Population displacement	***Implemented***: • Vaccination campaign ***Suggested***: Not mentioned
Njoh et al., 2022 [29]	Cameroon (Northwest Region), January 1st, 2020 to September 4th, 2021	COVID-19 cases	• Massive internal displacement of the population • Looting and destruction of health facilities • Killing of healthcare workers, disruption of the healthcare system in the region • Challenges related to delivering vaccines in security compromised areas	***Implemented***: Not mentioned ***Suggested***: • Scale- up COVID-19 vaccination • Innovative approaches adapted to the local context including community participation at every level of the vaccine rollout • Carrying out vaccination at transit points such as bus stations and refuge sites
Uwishema et al., 2022 [31]	Ukraine, From 24 February 2022	Population of Ukraine	• Shattered healthcare infrastructure, wreaked primary healthcare facilities • Patients who managed to make it to hospitals encountered severe constraints such as power outages and oxygen shortages • Mass migrations • People sought refuge in closed subway systems or migrated to more shielded places	***Implemented***: Not mentioned ***Suggested***: • Rebuilding of the broken healthcare System • NGOs, doctors and front-line workers should extend their support and supply vaccines and medicine • Preparation of standard medical facilities, i.e. medications, equipment, medical military personnel and a combat support hospital with intensive care capacity. • Education of military personnel and civilians on infectious diseases, personal protective measures, immunisations, chemoprophylaxis and surveillance
Quinn et al., 2021 [30]	Ukraine, 2020 - publication date, April 2021	Population of Ukraine	• Lack of health-related infrastructure and health-care staff that were forced to migrate to safe locations • Disaster response was further hindered as resources were allocated to warfighting efforts against an invading and occupying force • Primary health-care services were destroyed during the Russian invasion • Reduced access to basic primary prevention for the paediatric population • In occupied territories health-care system was poorly equipped • Many regional medical centres lacked COVID-19 testing in the beginning of the outbreak • Insufficient logistical equipment of hospitals; • Lack of qualified specialists on infectious diseases, virology, epidemiology • Lack of surveillance and appropriate testing labs • Lack of adequate personal protective equipment • During the winter period from 2020 to 2021, climate problems were identified and complicated COVID-19 detection and diagnosis	***Implemented***: • Social distancing • Face masks • Handwashing • Isolation • Quarantine • Limited movement • Limited traveling abroad especially to countries where COVID-19 cases are confirmed • Diagnostic test kits at checkpoints across state borders/ increased testing services at ports of entry • Financing of the production of RT-PCR tests by the Ukrainian Institute of Molecular Biology and Genetics of the National Academy of Sciences • Self-isolation of troops • Military medical clinical centres and deployed mobile hospitals were supported for COVID-19 prevention and treatment ***Suggested***: • The medical readiness system in Ukraine will need to create reserves of medical equipment, medicines, medical devices, personal protective equipment, disinfectants, and capacity building and training • Surveillance and testing policies not only for severe cases but should include milder cases and asymptomatic infections • Broad access to accurate testing • International coordination, knowledge and information sharing between states/ international partners for rapid implementation of containment, mitigation, treatment, and rapid vaccine rollout options • Step up capacity building efforts to train and supply Ukrainian epidemiologists and laboratories to handle the diagnostic and biostatistics requirements for responding to any infectious disease outbreak and integration biosurveillance, antimicrobial resistance • Strict prioritisation in allocation of resources, beds, and medical staff • Interoperability with international partners must be increased and expanded upon across all domains of battle and disaster response

Abbreviations: COVID-19 = Coronavirus Disease of 2019, NGOs = Non-governmental organisations, RT-PCR = reverse transcription polymerase chain reaction

The reported emergency control measures included social distancing, face masks, hand washing, isolation, limited movement and travelling, acquisition of diagnostic test kits for COVID-19 detection at checkpoints across state borders, self-isolation of troops, and the deployment of mobile hospitals and military medical centres for COVID-19 prevention and treatment [30]. In addition, immediate financing was provided to the Institute of Molecular Biology and Genetics of the National Academy of Sciences of Ukraine for the production of self-tests [30].

Suggestions for mitigating SARS-CoV-2 transmission included enforcing national policies with internationally accepted guidelines and tools adapted to conflict situations [26] and rebuilding healthcare systems [31]. It was further suggested that NGOs, doctors and front-line workers should extend their support by supplying vaccines and medicine and that standard medical facilities should be prepared with medications, equipment, medical military personnel, and a combat support hospital with intensive care capacity [31]. Other suggested preparedness strategies included training of health facility staff [26, 28], disease awareness programmes for civilians [26, 28, 31] and for military personnel [31], establishment of isolation spaces [28], and the scale-up of COVID-19 vaccination [29]. It was additionally suggested that surveillance and testing policies should not be restricted to severely hospitalized patients but should include milder cases and asymptomatic infections and that access to accurate rapid tests should be broad [30]. The allocation of resources, beds, and medical staff was recommended to be on strict prioritization [30]. Finally, information sharing of data and interoperability with international partners was stressed [30].

### Tuberculosis

With this systematic review, five studies were identified that reported on tuberculosis (TB) in conflict settings between January 2000 and October 2023 [32–36] (Table 4). Increased TB incidence was reported in Ethiopia [32], South Sudan [34], and Nigeria [33], following armed conflict, and recently appeared in Ukraine, Russia and neighbouring countries due to the displacement of citizens [35, 36].

Delay in the diagnosis of TB patients and self-treatment prior to diagnosis have been associated with increased transmission and morbidity [32]. The armed conflicts in Ethiopia disrupted the healthcare system and economic resources were diverted to priorities other than health needs, this meant that patients were unable to seek prompt TB care resulting in diagnostic delay and hampered TB control efforts [32]. In South Sudan, key challenges resulting from civil unrest that contributed to TB transmission included the limited number of healthcare providers, the interruption of treatments because travel was impossible, and the relocation of people [34]. During the war in Ukraine and Russia, internal displacement of citizens resulted in the dispersal of drug-resistant Mycobacterium tuberculosis to affected and neighbouring countries. The heightened risk of interrupted treatment during war contributes to an increased likelihood of drug resistance and treatment failure [35]. In addition, the war resulted in a large population movement fleeing Ukraine to reach France among which TB cases and consequently the spread of the TB [36] Finally, Adamawa State experienced several years of violence with severe disruption of public health activities including TB services and a massive population displacement [33]. The study shows that years and places of higher conflict were associated with lower TB notifications [33]. The decrease reflects the displacement of the populations to other locations that were considered safer, the limited or no access to TB health services due to displacement, the general disruption of TB services along with the reduced number of healthcare staff [33].

Suggested measures for TB control in conflict zones were the expansion of user-friendly directly observed short-course treatment (DOTS), the establishment of early TB detection training programmes for community health workers [32], and population-target risk communication activities [33]. Importantly, international organisations providing health services should be given unconditional access to conflict zones [32] while the global health community should be ready to step up efforts to detect and treat drug-resistant and drug-susceptible TB, as well as to strengthen screening initiatives for TB prevention and treatment in migrants and close contacts [35]. Finally, it was noted that a pre-established well-organised network of TB centres such as the CLAT network is effective in the case of sudden mass migration from a high TB incidence country [36].

**Table 4:** Impact of conflict on Tuberculosis outbreaks

*Author*	*Country, Setting, Timeframe*	*Population*	*Conflict to Disease Pathways*	*Prevention and Preparedness Strategies Suggested/Implemented*
Gele and Bjune, 2010 [32]	Somali Regional State of Ethiopia, Population from the tuberculosis management units in the Jigjiga and Shinile zones of the Somali Regional State, June – September 2007	TB Patients in the intensive phase of treatment	• Large number of military conflicts may impact TB control programmes by interfering with the goals of identifying and curing TB patients. • Armed conflicts may not only fuel TB epidemics by escalating poverty and malnutrition, and thereby increase the number of TB susceptible individuals, but also cause diagnostic delays by deterring infectious TB patients from seeking prompt diagnosis and treatment. • Access to health care is often limited by the lack of security. • Armed conflicts hamper TB control efforts not only by disrupting the health system but by diverting economic resources to priorities other than health needs.	***Implemented***: Not mentioned ***Suggested***: • Improve the access to TB diagnosis and treatment. • Sustainable political commitment for the implementation of successful TB control programmes. • Expansion of user-friendly directly observed therapy short-course (DOTS) in the conflict zone. • Establishment of training programmes for community health workers for early detection of TB patients. • International organisations providing health services should be given unconditional access to conflict zones.
Boyong et al., 2018 [34]	Wau, South Sudan, Wau Teaching Hospital in armed conflict in South Sudan, January – February 2016	Suspected tuberculosis cases at Wau Teaching Hospital	• The city of Wau had been at the centre of conflict and the Wau Teaching Hospital, the only hospital of this size in a 350-km radius serving approximately 3 million people, had critical shortage of professional health workers (1.5 physicians and two Nurses/Midwifes were available for every 100,000 citizens) • Patients travelled long distances, which were interrupted by gunfights, to seek TB medical attention • Interrupted treatments because it was impossible to travel. • Displacement of people	***Implemented***: Not mentioned ***Suggested***: Not mentioned
Pembi et al., 2020 [33]	Adamawa State, North-east Nigeria, Adamawa State 2010–2016	Tuberculosis cases	• Adamawa State experienced several years of violence, with a severe disruption of public health activities and a massive population displacement • TB services are at risk in areas with political disruption and conflict • Years and places of higher conflict were associated with lower TB notifications. • Displacement of the populations to other locations that were considered safer • Refugees and IDPs with limited access to TB health services, or no access due to transport • Disruption of TB services • Reduced numbers of health staff	***Implemented***: • TB Reach-funded project in all areas during which TB risk messages were broadcasted through jingles in the local radios • Health workers were re-trained on TB identification, diagnosis, treatment and follow-up • 402 community volunteers were engaged to boost awareness and reporting of cases • Introduction of GeneXpert testing increased awareness on TB ***Suggested***: Not mentioned
Dahl et al., 2022 [35]	Ukraine and Russia	Population of Ukraine and Russia	• The internal displacement of citizens, especially within Ukraine but also in Russia, and migration of war refugees has large consequences including the dispersal of drug-resistant Mycobacterium tuberculosis in both the affected and neighbouring countries, which are presently facing an un-precedented flow of refugees. • Initiating and maintaining a course of anti-TB therapy during war, or during migration, is undoubtedly also associated with a higher risk of inappropriate or interrupted treatment and, followingly, an increased likelihood of drug-resistance, treatment failure and death.	***Implemented***: Not mentioned ***Suggested***: The international health community must be prepared to intensify the capacity of detection and treatment of both drug-susceptible and drug-resistant TB, and to strengthen screening programmes for TB prevention and treatment of active disease among migrants and close contacts of people with TB, ultimately to diminish the impact of the ongoing conflict and its future consequences for global health.
Guthmann et al., 2023 [36]	Ukraine and France 2022–2023	Population of France	• The spread of TB due to the war and the large population movement fleeing Ukraine to reach France among which TB cases	***Implemented***: Active CXR screening to detect TB cases among the displaced population ***Suggested***: Pre-established well-organised network of TB centres such as the CLAT network in case of sudden mass migration from a high TB incidence country. Increasing effectiveness of the existing strategy may require further well-trained man-power and financial support, both likely not being readily available.

Abbreviations: CLAT = Réseau National des Centres de lutte antituberculeuse, CXR = chest X-ray, DOTS = Directly Observed Treatment Short-course, IDPs = internally displaced people, TB = tuberculosis.

### Ebola virus disease

The current systematic review identified five studies on Ebola virus disease and conflict published between January 2000 and October 2023 [37–40] (Table 5). The Ebola epidemic in the Democratic Republic of Congo (DRC) [37−40] occurred in the midst of an active armed conflict, geopolitical volatility and with a million displaced people [31]. The conflict in the region was associated with inhibited case detection [39, 40], delayed reporting of the outbreak [37, 40], delayed time to isolation [40], deteriorating security [39], dampened vaccine deployment [40], and limited follow-up [40] especially with people in the zone of violence [37] all of which led to an increase in transmission that was attributable primarily to the organised attacks by armed groups targeting healthcare providers and Ebola treatment centres [39, 40] and the population’s increasing distrust of the response effort [38–40] that impedes information-sharing and cooperation.

**Table 5:** Impact of conflict on Ebola outbreaks

*Author*	*Country, Setting, Timeframe*	*Population*	*Conflict to Disease Pathways*	*Prevention and Preparedness Strategies Suggested/Implemented*
Nakkazi 2018 [37]	DR Congo, North Kivu in DR Congo, 1 August 2018–19 August 2018	People of North Kivu in DR Congo	The conflict played a role in delaying the detection of the outbreak for 3 months	***Implemented***: • The DR Congo Ministry of Health coordinated and oversaw vaccination roll­out and followed the WHO emergency use assessment and listing procedure protocol. • All contacts and their contacts should be vaccinated • The DR Congo needed help from partners to respond and control this outbreak ***Suggested***: Not mentioned
Gostin et al., 2019 [38]	DRC, People in DRC during armed conflict, Between October 28 and November 26, 2018	People in DRC	• The Ebola epidemic occurred within active armed conflict and geopolitical volatility, including a million displaced persons • Infection of healthcare workers • Community distrust is deep after decades-long humanitarian crises, impeding information-sharing and cooperation	***Implemented***: • Contact tracing • Medical isolation • Ring vaccination • Investigational treatments • Foreign health workers, nongovernmental organisations, and UN agencies had been leading an energetic international Ebola epidemic response, alongside local personnel who offered experience and linguistic and cultural awareness • World Bank dispatched financing, while US-supported vaccines, therapies, and laboratory/epidemiology capacity- building were proved essential ***Suggested***: • Responders need greater capacity in surveillance, data analysis, laboratories, and clinical response, particularly experienced personnel to work with local leaders to build community trust and communication. • Increased security and ensuring safe humanitarian operations • The UN Security Council should mobilize high-level political attention and resources for the Ebola response • External partners should develop a plan to deploy public health personnel such regions • There should be an increase in funding to enhance local response capabilities • A transparent framework for responding to epidemics in conflict zones should be developed • Sustainable funding for national action plans for health security and a plan to safeguard public health action in conflict zones should be created
Ilunga Kalenga et al., 2019 [39]	DRC, 28 July 2018–7 May 2019	Residents of DRC	• Organised attacks by armed groups targeting response teams and Ebola treatment centres, • Deteriorating security • Population’s increasing distrust of the response effort	***Implemented***: • 55 million entry and exit screenings • Real-time epidemiologic surveillance of contacts • Provision of safe and dignified burials for most deaths • Vaccination of high-risk people • Medical treatment including four investigational therapies • Rapid rollout of vaccine • Border screening • Rapid decontamination of facilities where cases have been identified • Health care facilities and key sites (schools, public offices, and transit points) equipped with training, infection prevention and control equipment (including personal protective equipment), and essential consumables such as chlorine, soap, and water ***Suggested***: Not mentioned
Wells et al., 2019 [40]	DRC, SIR model for the Ebola outbreak in DRC, 30 April 2018–23 June 2019	Population of Congo	• This period of civil unrest inhibited case detection and delayed reporting of the outbreak • Conflict events were found to reverse an otherwise declining phase of the epidemic trajectory with disruptive events found to extend the average time from symptom onset to isolation and dampen vaccine deployment and increase transmission • Case identification and containment of Ebola was even more difficult in areas that were too dangerous for health workers to enter or work (only 20% of contacts were traced) • Several conflict events including attacks on ETCs or healthcare workers and healthcare workers protests had direct impact on the public health response • Mistrust of the government and the public health response among civilians compounded hostility • Healthcare providers became the target of violence	***Implemented***: Not mentioned ***Suggested***: • Integrating humanitarian work in the response • Community engagement is needed to improve trust among the residents • Engendering trust among locals early in an outbreak through community engagement • Fundamental to ensure that frontline workers providing treatment, conducting contact tracing, and distributing vaccines can work efficiently

Abbreviations: ETCs = Ebola treatment centers, UN = United Nations, US = United States, WHO = World Health Organisation

In response, the DRC Ministry of Health collaborated with health workers from NGOs and UN agencies and, alongside with the linguistic and cultural awareness of local personnel, implemented contact tracing [38], medical isolation [38], exploratory therapies [38], ring vaccination [37−39], entry and exit screenings at key points [39], real-time epidemiologic surveillance of contacts [39], provision of safe and dignified burials [39], and medical treatment [39]. Additional measures that aided the outbreak’s containment were the rapid decontamination of facilities with identified cases and the distribution of infection prevention and control equipment to healthcare facilities and strategic areas (schools, public offices, and transition points) [39].

Suggested preparation strategies included engaging the community to build trust among residents [38–40], ensuring the safety of frontline workers that provide treatment, conducting contact tracing, and distributing vaccines [38–40]. In addition, the significance of increased security and expanded capabilities in laboratories, surveillance, data analysis, and clinical response were emphasised [38]. Finally, the necessity of a transparent framework for responding to epidemics in conflict zones was acknowledged, which should be supported by national action plans to safeguard public health action in conflict zones [38].

### Poliomyelitis

The current review identified five studies published between January 2000 and October 2023 on poliomyelitis and conflict [41–45] presented in Table 6. According to a study that collected data from countries affected by conflict and experiencing polio outbreaks between 2011 and 2014, polio was more common in countries with political conflict and instability [42]. People faced a lack of access to clean water, as well as deteriorating sanitation and living conditions as a result of conflict and political instability, which aided polio transmission significantly [42]. Furthermore, polio incidence was found to be spatially associated with violence [as represented by the location of Improvised Explosive Devices (IEDs)] in Afghanistan [44]. The high-risk districts had a statistically significant greater mean number of IEDs compared to non-polio high-risk districts [44]. According to the authors, violence in the region leads to reduced rates of polio vaccination and disruption in vaccine coverage, which in turn is responsible for increased polio incidence [44]. It was reported that vaccination campaign workers and public health workers had been directly targeted by armed groups, with abductions and murders. The Afghani government and international agencies had been forced to suspend operations or delay subnational immunisation days in some regions. The response was challenged from the combined impact of a government transition, a depressed economy, droughts, floods, food insecurity, displacement, and severe gaps in delivery of health services [45].

**Table 6:** Impact of conflict on Poliomyelitis outbreaks

Author	Country, Setting, Timeframe	Population	Conflict to Disease Pathways	Prevention and Preparedness Strategies Suggested/Implemented
Cetorelli 2015 [41]	Iraq, October & November 2000, February to March 2006, and 2011	Children under 5 over the three timepoints in Iraq	Broader war induced deterioration in the country’s healthcare capacity and vaccine rollout	***Implemented***: Not mentioned ***Suggested***: Promoting institutional deliveries and ensuring adequate vaccine availability in primary health facilities
Akil and Ahmad, 2016 [42]	Pakistan, Afghanistan, Nigeria, Syria, Iraq, Cameroon, Equatorial Guinea, Ethiopia, Kenya, and Somalia, Conflict-affected countries, 2011–2014	Aggregated country-level data on WPV cases from conflict-affected countries	Polio was higher in countries with political conflict and instability • Poor infrastructure • Population movement • Mistrust by local community in the national authorities regarding immunisation, thus increased rates of unvaccinated children • Hard-to-reach populations • Lack of access to clean water • Collapsing sanitation and living conditions	***Implemented***: • WHO mandated polio vaccination for all individuals travelling to or from Pakistan, Syria, and Cameroon • In 2013, the GPEI launched a five-year all-encompassing plan for completely eradicating polio, a strategic plan that clearly outlines measures for eliminating polio in its last strongholds and for maintaining a polio-free world ***Suggested***: For displaced families and others in these high-risk areas: • Immediate health care • Clean water • Increased nutritional measures • Better sanitation • Easy access to healthcare • GIS maps may help to identify areas with high rates of polio and to predict the possibility of movement of the virus to neighbouring countries to assess virus origins and the current virus movement
Norris et al., 2016 [44]	Afghanistan, 2004–2009	Data collected in Afghanistan from 2004 to 2009	• Violence leads to reduced rates of polio vaccination, which is, in turn, responsible for increased polio incidence • Vaccination campaign staff and public health staff were being directly targeted by armed groups, with abductions and murders • Conflict creates a lack of public trust in the governmental and international organisations that run vaccination campaigns. Without trust, a successful vaccination programme is difficult • Violence can damage infrastructure, cause suspension of vaccination activities, and influence the behaviour of whole communities	***Implemented***: The GPEI, a project of the WHO, coordinated the efforts of the Afghani government, UNICEF, and various NGOs to eradicate polio. The GPEI national team was responsible for policy, planning, and vaccine supply, while provincial teams were responsible for implementation, supervision, and monitoring of programme activities: • Organised 1,251 vaccination sites and over 2,700 vaccinators were organised to provide routine services • Organised supplemental immunisation activities including national and subnational immunisation days • Conducted “mop-ups:” children in the vicinity of a polio outbreak were revaccinated ***Suggested***: • Support polio vaccine distribution efforts in communities exposed to violence • Take all available measures to avoid entangling the polio vaccination campaign in political dynamics of the armed conflict • Direct negotiation to convince anti-government groups to allow safe passage of health staff through opposition-controlled regions
Al-Moujahed et al., 2017 [43]	Syria, Mid 2013 and after	Population of Syria	• Systematic assaults on healthcare in politically unsympathetic areas resulted in the collapse of the healthcare system in oppositionheld territory • Severe damage to hospitals, public health centres, and ambulances • Deliberate target and persecution of healthcare personnel • Emigration of healthcare personnel from both government and nongovernment territory as a result of the conflict • Severe lack in medications and preventative services • Children in some besieged and opposition-controlled areas were missing vaccinations • Sharp decline in the overall vaccination coverage to only 50% in 2015 • Destruction of the country’s infrastructure • Economic shrinkage • Severe food and water insecurity • Inadequate sanitation	***Implemented***: The Polio Control Task Force (PCTF) was formed by eight Syrian and NGOs. This task force was able to successfully establish immunisation facilities, train about 8500 personnel from local communities and deliver vaccines to more than 1.4 million children across seven governorates in northern and eastern Syria, areas inaccessible to WHO. ***Suggested***: • Important to increase international surveillance and international financial and logistical support for vaccine and immunisation of the population especially in conflict-torn countries • Adequately support and fund the front-line NGOs that are implementing the delivery of medical and humanitarian aid in Syria and to refugee populations in neighbouring counties • Agencies involved in global health to be able to operate impartially, from governments and all military actors involved during conflicts and enabled to provide necessary and efficient medical and humanitarian relief for civilian from governments during conflicts in order to provide adequate and efficient medical and humanitarian relief for civilians
Mohamed et al., 2022 [45]	Afghanistan January 2021- September 2022	Population of Afghanistan Poliomyelitis	• Polio eradication efforts in Afghanistan were challenged by a complex humanitarian emergency resulting from the combined impacts of a rapid government transition and a depressed economy, droughts, floods, food insecurity, displacement, and severe gaps in delivery of health services • Unreachable children for vaccination due to insurgency	***Implemented***: • In 2020 when Afghanistan began to report both cVDPV2 and WPV1 polio cases, the Global Polio Eradication Initiative authorised the use of tOPV for outbreak response. • In 2022 the program reached 3.5–4.5 million children previously unreachable because access was prevented by the insurgency • Lot quality assurance sampling (LQAS) surveys were conducted to assess SIA quality • Development of Acute Flaccid Paralysis and also environmental surveillance with the systematic sampling and virologic testing of sewage sites • Genomic sequence analyses were performed to assess cross border transmission between Afghanistan and Pakistan [45] ***Suggested***: Not mentioned

Abbreviations: cVDPV2 = type 2 circulating vaccine-derived poliovirus, ETCs = Ebola treatment centers, GIS = Geographic Information System, GPEI = Global Polio Eradication Initiative, NGOs = non-governmental organisations, PCTF = Polio Control Task, Forcem, SIA = supplementary immunization activities, tOPV = trivalent OPV, UN = United Nations, UNICEF = United Nations Children’s Fund, US = United States, WHO = World Health Organisation, WPV = wild poliovirus, WPV1 = endemic wild poliovirus type 1

In Syria, poliomyelitis reappeared in mid-2013 during the civil war [43]. Reported conflict-to-disease related factors included the collapse of the healthcare system and infrastructure, decline in the economy, and shortages of food, water, and inadequate sanitation [43]. The emigration of healthcare personnel from both government and nongovernment territory due to conflict further affected the country’s healthcare system [43]. Moreover, a severe lack of basic medications and preventative services had ensued, including a sharp decline in the overall vaccination coverage to only 50% in 2015 [43]. Finally, in Iraq children who had been exposed to war were over 20% points less likely to receive neonatal polio immunisation compared to children who had not been exposed [41]. According to the authors, the decline is part of a broader war-induced deterioration of routine maternal and new-born health services [41].

In response, the World Health Organization (WHO) mandated polio immunisation for all travellers to and from Pakistan, Syria, and Cameroon and suggested travel vaccinations for Afghanistan, Nigeria, and other nations as a preventive measure [42]. Additionally, the Global Polio Eradication Initiative (GPEI), which coordinated the actions of the Afghan government, UNICEF, and NGOs, represented a significant polio mitigation effort through which vaccination sites, vaccinators, and supplemental immunisation activities were organised, and “mop-ups” (i.e. revaccination of children close to a polio outbreak) were carried out [44]. Moreover, in 2020 the GPEI authorised the use of trivalent OPV (tOPV) for outbreak response, with supplementary immunization activities carried out throughout 2021 and 2022 outbreak response [45]. In 2022 the program reached 3.5–4.5 million children that were previously unreachable because the insurgency prevented access [45]. The development of Acute Flaccid Paralysis took place and also environmental surveillance with the systematic sampling and virologic testing of sewage sites [45]. Finally, genomic sequence analyses were preformed to assess cross border transmission between Afghanistan and Pakistan [45]. Regarding Syria, Syrian and regional NGOs, in an effort to control the outbreak, established vaccination facilities, trained local personnel, and delivered vaccines to children in areas inaccessible to WHO [43].

The control of polio and other infections in impoverished, conflict-ridden areas was suggested that may be improved by providing displaced families and those in high-risk areas with urgent care in the form of clean water, increased nutritional measures, improved sanitation, and easy access to health care and vaccinations [42]. Additionally, Geographic Information System (GIS) maps presenting the recent virus origin and the current virus movement may help to identify areas with high rates of polio and to predict the possibility of movement of the virus to neighbouring countries, thus guiding preventative measures [42]. Furthermore, the lack of polio-licenced laboratories or the lack of access to laboratories in conflict zones was noted, necessitating international surveillance to be strengthened [43]. Finally, financial and logistical international support for vaccine and immunisation of the population in conflict-torn countries is needed [43]. The authors emphasised that during conflicts, WHO, UNICEF, and UN agencies involved in global health should be supported, funded and allowed to function independently of governments to provide necessary medical and humanitarian relief for civilians [43]. Also, in order to ensure uninterrupted immunisation coverage and thus successful eradication of polio in conflict zones, direct negotiations with anti-government organisations should be conducted [44].

### Malaria

The current review identified two articles on malaria prevalence in conflict-affected areas of Timor-Leste and Sub-Saharan Africa that were published between January 2000 and October 2023 [46, 47], presented in Table 7. During the 2006 Timor crisis, gang fights and street violence ensued, over 3,000 homes burned down mostly in the capital city, Dili and 15% of the country’s population was displaced [46]. The IDPs sought refuge in camps, churches, convents and schools, with some displaced from Dili, to districts [46]. In Dili, more than 60 camps were established to provide temporary shelter for displaced people [46]. Breakdown of health services and of malaria control programmes, movement of people from low to high transmission areas, and environmental deterioration encouraging vector breeding, such as rainy seasons are factors that contribute to the increase of morbidity and mortality due to malaria [46]. The authors argue that the timing of the crisis which occurred at the end of the rainy season along with the early malaria interventions which covered treatment, massive insecticide-treated nets [48] distribution with emphasis to pregnant women and children under five, vector control, surveillance and health promotion for IDPs possibly prevented major Malaria outbreaks in the area [46]. It was recommended that future malaria intervention responses be planned beyond the IDP camps and adequate resources and expertise be made available to ensure a whole-city approach [46].

**Table 7:** Impact of conflict on Malaria, Leishmaniasis, Measles, Dengue, Diphtheria and ABM outbreaks

*Author*	*Country, Setting, Timeframe*	*Population/Type of Infectious Disease*	*Conflict to Disease Pathways*	*Prevention and Preparedness Strategies Suggested/Implemented*
Martins et al., 2009 [46]	Dili and four other districts: Aileu, Baucau, Ermera and Lautem, IDP camps and health facilities, September –November 2006	Key informant interviews (*N* = 30), document reviews, focus group discussions (*N* = 3) and malaria morbidity data Malaria	• Conflict led to the displacement of thousands of people and interrupted routine malaria service programmes • Movement of people from low to high transmission areas, and environmental deterioration encouraging vector breeding, such as rainy seasons aid malaria transmissions	***Implemented***: Collaboratively and rapidly organised interventions for IDPs covering: • treatment, • insecticide treated net distribution, with priority to pregnant women and children under five • vector control, • surveillance ***Suggested***: Intervention response must be planned beyond the IDPs alone, and adequate resources and expertise should be made available to assure a whole-of-city approach
Sedda et al., 2015 [47]	Sub-Saharan African countries, 1997–2010	General population Malaria	• The impact of conflicts on the prevalence of malaria is stronger in the presence of violent events (e.g., violence against civilians and riots/protests).	***Implemented***: Not mentioned ***Suggested***: Maintenance of intervention coverage and provision of healthcare in conflict situations to protect vulnerable populations
Alawieh et al., 2014 [49]	Lebanon, January 2013 – March 2014	Lebanese, Syrian refugees, and Palestinian refugees Leishmaniasis	• Massive and rapid increase in the arrival of Syrian refugees • Infiltration of a high number of Syrian refugees in dense concentrates to different regions of Lebanon without restriction to designated camps. • Limited access to treatment and the absence of well-trained healthcare professionals on this topic	***Implemented***: The LMOPH to contain the spread of infection: • sprayed pesticides to kill the vector • provided free treatment and diagnosis for emerging cases, • distributed medications free of charge to the different primary care centres, • monitored of disease activity, • trained physicians and health care workers on disease symptoms, raising their index of suspicion, • educated the Lebanese public and Syrian refugees about the disease symptoms and how to seek medical advice and treatment. • assigned new centres for Leishmania detection and treatment in all Lebanese hospitals, including those in rural areas and near refugee camps. ***Suggested***: The coordinated efforts and cooperation among governmental departments, international agencies, local authorities, medical associations, and NGOs are critical for containing any outbreak.
Youssef et al., 2019 [50]	Latakia city, Syria, 2008–2016	Population of Latakia city Leishmaniasis	The 2011 Syrian conflict: • Displaced more than 6.5 million people • Devastated the Syrian healthcare infrastructures, severely damaging 60% of the Syrian hospitals, and greatly reducing the pharmaceutical production capacity of the country. • Crowding of the “safer Syrian cities” and their healthcare facilities, and displacement of a large population from leishmaniasis-endemic areas • Potentially exposed younger males enrolled in the army who would transfer the parasite.	***Implemented***: The LMOPH initiated a control campaign following the 2013 outbreak in the Lebanese refugee camps that included: • Vector control • Early detection • Free treatment of leishmaniasis cases A similar campaign was implemented in the Latakia governorate to counter the leishmaniasis outbreak after 2013 which consisted of: • Vector control mainly indoor residual spraying • Early detection and treatment • Public education. ***Suggested***: • Improving the living circumstances in sites with high population densities, • Enforcing better healthcare services • Activating surveillance, early diagnosis, vector control, and public education
CDC 2004 [51]	Sudan, Darfur, 2004	Children aged 9 months – 5 years Measles	• Darfur experienced civil conflict during the previous year, resulting in the internal displacement of approximately one million residents and an exodus of an estimated 170,000 persons to neighbouring Chad. • The conflict left a vulnerable population with limited access to food, health care, and other basic necessities. • Measles vaccination coverage had been adversely affected	***Implemented***: State ministries of health and various NGOs conducted: • Vaccination campaigns in IDP camps and neighbouring communities, targeting children aged 9 months–5 years; these campaigns vaccinated approximately 80,000 children. • Clinics were established in IDP camps to vaccinate current and incoming residents. • Vaccination using a combination of fixed posts and outreach immunisation teams, • Use of checklists to monitor vaccination sessions, • Social mobilisation activities, and • Surveillance for adverse events after vaccination • Rapid convenience surveys were used to monitor coverage in hard-to-reach areas. • At the state level, meetings were held at the end of each working day to review progress and address problems. • Tally sheets were used to monitor campaign coverage, and data were sent to the federal level for compilation and analysis. • Vaccination sites included fixed centres, temporary posts, and mobile teams. ***Suggested***: Not mentioned
Babakura et al., 2021 [52]	Nigeria, Borno State, 2017–2018	Measles surveillance data among children, Nigeria Measles	• The majority of the LGAs were not fully accessible for optimal conduct of the measles immunisation campaign • Children in inaccessible areas were denied access to immunisation services. • Inability to implement RES strategy to deliver measles vaccine to partially accessible areas because of escalation in insurgency during the time frame and the prioritisation of the military and joint task forces on addressing the security risk over delivering health commodities • Aggravation of factors related to disease transmission like the mass movement of people between IDP camps and host communities which may introduce transmission of measles	***Implemented***: Organised vaccination campaigns based on the accessibility mapping of each area developed by the government and partners. The immunisation strategies included: • Fixed posts (where the team was based at the health facilities) and temporary posts (located at strategic areas of the communities such as schools, markets, places of worship etc.). • The RES strategy which was implemented in some partially accessible parts of the State involved the deployment of vaccination teams with security cover by the Military or armed local vigilante referred to as the Civilian Joint Taskforce • Combined Human and Animal Vaccinations teams were organised targeting nomadic populations. ***Suggested***: • The strategy of Reaching Inaccessible Children was adopted to reach eligible children in security-compromised areas by leveraging the military personnel to conduct vaccination, but the plan was suspended and, thus, not implemented. • The engagement with the military must continue and be strengthened to ensure increased collaboration with security forces • The re-establishment of holding camps and vaccination posts at all entry points into IDP camps and host communities to ensure all new entrants are screened and vaccinated with Measles vaccines
Alghazali et al., 2019 [53]	Taiz, Yemen, Hospital and medical centres in Taiz, 2016	Patients with clinical suspected dengue in hospitals and medical centres in Taiz, Yemen Dengue	• The civil war: • caused widespread destruction to public health infrastructure • displaced > 2.2 million persons into living in cramped shelters with poor hygiene and inadequate healthcare support • created numerous potential mosquito-breeding sites, such as open water storage containers, areas with inadequate drainage, discarded plastic containers in which water accumulates, and puddles of water	***Implemented***: Not mentioned ***Suggested***: Not mentioned
Weil et al., 2021 [54]	Bangladesh, MSF diphtheria treatment centres located in Balukhali and Jamtoli camps, 2017–2019	Patients with symptoms of diphtheria Diphtheria	• A massive influx of approximately 630 000 FDMNs • Makeshift settlements in and around established refugee camps in Bangladesh where the first suspected diphtheria case was reported • Over 800 suspected cases reported in the area by mid-December 2017	***Implemented***: Not mentioned ***Suggested***: Not mentioned
Al-Samhari et al., 2023 [55]	Yemen 2014–2020 all children aged < 5 years admitted to all the nine sentinel hospitals in Yemen 2014–2020	All children aged < 5 years admitted to all the nine sentinel hospitals in Yemen 2014–2020 Acute Bacterial Meningitis (ABM)	• Ddisplacement of 4.3 million people, • 20.1 million people unable to access healthcare and > 20.7 million people in need of humanitarian aid • With the continuation of the conflict, the vaccination coverage rate dropped • The major government maternal and children’s hospital was repeatedly attacked and subject to armed incursions that resulted in damage not only to the medical infrastructure but also resulting in many patients leaving against medical advice	***Implemented***: Not mentioned ***Suggested***: • Immunisation Program on Immunization through sustainable investments in war-damaged infrastructure and providing decentralised finances are prerequisites • More serotype/group data for ABM patients are needed to better understand the prevalence of specific pathogen strains across Yemen

Abbreviations: ABM = acute bacterial meningitis, FDMNs = forcibly displaced Myanmar nationals, IDP = internally displaced people, LGAs = local government areas, LMOPH = Lebanese Ministry of Public Health, MSF = Médecins Sans Frontières centres, NGOs = non-governmental organisations, RES = Reaching Every Settlement

The second study examined the link between conflicts and variations in the *Plasmodium falciparum* parasite in Sub-Saharan African countries from 1997 to 2010, during which a significant number of armed conflicts occurred [47]. The duration of conflicts, the distance from conflicts, the number of conflicts, and the level of violence associated with the conflicts were found to be major factors that explained the prevalence of malaria [47]. More specifically, locations affected by a larger number of longer and closer conflicts with significant amounts of violence and deaths were more likely to see an increase in *P. falciparum* prevalence [47]. Moreover, decreased post-conflict *P. falciparum* parasite rate was associated with conflicts without violence against civilians, without violent transfer of territory or battles with change of territory, and without riots/protests [47]. The maintenance of intervention coverage and provision of healthcare in conflict situations to protect vulnerable populations was suggested [47].

### Leishmaniasis

This systematic review identified two studies on leishmaniasis displayed in Table 7 [49, 50]. Leishmaniasis outbreaks occurred in the conflict-affected regions of Lebanon [49] and Syria [50]. The outbreak in Lebanon was reported to be a result of the Syrian crisis and the consequent influx of Syrian refugees [49]. The massive and rapid increase in the arrival of Syrian refugees, and their large-scale movement to different regions of Lebanon, without allocation to designated camps, along with the limited access to treatment and the absence of well-trained personnel exacerbated the spread of the parasite [49]. The Lebanese Ministry of Public Health (LMOPH) to contain the spread of infection implemented the following measures: sprayed pesticides to kill the vector, provided free treatment and diagnosis for emerging cases, distributed free medications to the different primary care centres, and monitored the disease activity [49]. Additionally, medical and healthcare professionals received training on disease symptoms, and the Lebanese people and Syrian refugees received education on disease symptoms and medical treatment [49]. Furthermore, the government established new Leishmania detection and treatment units in all Lebanese hospitals, mainly in rural areas and near refugee camps [49]. The authors note that the measures taken by the LMOPH are key to any health emergency response, but require the cooperation of other concerned parties to ensure success [49]. The importance of coordinating efforts among various governmental departments, international agencies, local authorities, medical associations, and NGOs for containing similar outbreaks in Lebanon, or any other country in the region is emphasised [49].

In Syria, the conflict displaced more than 6.5 million people causing a major refugee crisis, severely damaged 60% of Syrian hospitals, and greatly reduced the pharmaceutical production capacity of the country [50]. The destruction of healthcare infrastructure, the over-crowding of what was considered “safer Syrian cities” (such as Latakia) and their healthcare facilities, and the displacement of a large population from leishmaniasis-endemic areas enabled the transmission of the Leishmania parasite [50]. To control transmission, healthcare authorities initiated a campaign that consisted of vector control, early detection and treatment, and public education. Improving the living circumstances in sites with high population densities, enforcing better health-care services, and activating surveillance, early diagnosis, vector control, and public education is suggested as an integral part of any plan to successfully control and eliminate leishmaniasis [50].

### Measles

Two studies were identified on measles and conflict between January 2000 and October 2023 [51, 52](Table 7). Higher incidence rates of measles were reported in conflict-affected Darfur, Sudan [51] and in Borno state, Nigeria [52]. In 2003, Darfur experienced civil conflict that resulted in the internal displacement of approximately one million residents and an estimated 170,000 persons fleeing to neighbouring Chad [51]. The conflict left a vulnerable population with limited access to food, health care, and other basic necessities, which adversely affected measles vaccination coverage [51]. During March-April 2004 a measles outbreak among IPDs was reported in Darfur [51]. In response, the Federal Ministry of Health in collaboration with the UNs and NGOs conducted vaccination campaigns targeting children aged 9 months – 5 years to limit the transmission. Moreover, clinics were established in IDP camps to vaccinate current and incoming residents [51]. Despite these measures, measles virus transmission continued to occur both within the camps and in neighbouring communities [51]. Vaccination was organised using a combination of fixed posts and outreach immunisation teams, the use of checklists to monitor vaccination sessions, social mobilisation activities, and surveillance for adverse events after vaccination. In addition, rapid convenience surveys were used to monitor coverage in hard-to-reach areas [51]. Tally sheets were used to monitor campaign coverage, and data were sent to the federal level for compilation and analysis [51].

In Borno state, Measles vaccination campaigns were organised based on accessibility mapping of each area [52]. They organised both fixed locations of the healthcare team at health facilities and temporary locations at strategic areas of the communities (schools, markets, and places of worship) [52]. In addition, the Reaching Every Settlement (RES) strategy was implemented in some partially accessible areas and involved vaccination teams with security cover by the Military or armed local vigilante referred to as the Civilian Joint Taskforce [52]. For nomadic populations, combined Human and Animal Vaccination teams were organised [52]. Finally, the strategy of reaching Inaccessible Children was adopted to reach eligible children in security-compromised areas by leveraging the military personnel to conduct vaccination, but the plan was suspended by the military [52]. Although the above immunisation strategies were organised, the escalation in insurgency during the timeframe and the prioritisation of the military to address security issues over delivering health commodities compromised the vaccination campaign [52]. Most of the local government areas (LGAs) were not fully accessible and children remained trapped with no access to immunisation services [52]. Another reported factor related to the introduction and transmission of measles was the mass movement of people between IDP camps and host communities [52]. The authors recommended a continuous, stronger engagement with the military, re-establishment of holding camps, vaccination posts at all entry points into IDP camps and host communities to ensure screening and vaccination of new entrants [52].

### Dengue

Our systematic review identified one study reporting on the dengue outbreak in Taiz, Yemen in 2016 during the civil war [53](Table 7). According to the authors, the prevalence of dengue in Taiz increased markedly because of the ongoing civil war which damaged the public health infrastructure of the country [53]. More than 2.2 million people were displaced and moved into overcrowded shelters forced to live in unsanitary conditions with limited access to medical care [53]. The war led to the creation of numerous potential mosquito-breeding sites such as open water storage containers, areas with inadequate drainage, discarded plastic containers in which water accumulates, and puddles of water and resulted in propagation and transmission of the mosquito-borne dengue virus that was difficult to control [53]. No prevention or preparedness strategies were noted within this report.

### Diphtheria

Our review identified one study that investigated a diphtheria outbreak in Bangladesh among Myanmar nationals displaced due to political conflict [54]. As noted in Table 7, from August to December 2017, a massive influx of approximately 630,000 forcibly displaced Myanmar nationals created makeshift settlements in and around established refugee camps in Bangladesh where the first suspected diphtheria case was reported which resulted in over 800 suspected cases reported in the area by mid-December. No prevention or preparedness strategies were noted within this report [54].

### Acute bacterial meningitis (ABM)

Our review identified one study that assessed ABM outbreaks in Yemen before and during the civil war [55]. The study noted that the civil war reduced vaccination coverage and increased the prevalence of suspected cases, with areas which were more affected by civil war found to have the highest suspected prevalence and lowest vaccination coverage [55]. Overall, the ongoing war and the land–sea–air embargo imposed on Yemen eventually caused the vaccination coverage rate to decline [55].

### Studies assessing the impact of conflict on multiple infectious diseases

The current review identified and analysed six studies on multiple infectious diseases published between January 2000 and October 2023 [56–61] (Table 8). Higher incidence rates of several infectious diseases were reported in Iraq [56], in the Southwest region of Cameroon [57], in Syria and neighbouring countries [61], in Jordan [59], in Ukraine [58], and in DRC [60]. During the peak years of the war in Iraq, particularly during the US military surge (2007–2009), the incidence of infectious diseases increased significantly [56]. Iraq experienced four vaccine-preventable disease outbreaks: measles in 2009, mumps in 2004 and 2016, and rubella in 2004. These outbreaks were reported to have been attributed to deterioration of the infrastructure during the conflict, lower vaccine coverage and higher vaccine failure due to inappropriate vaccine handling, failure to maintain the cold chain, and improper administrative procedures [56]. The Cholera outbreak in 2008 was reported to have been facilitated by the war-related deterioration of water quality and sanitation. The rise in the incidence of Hepatitis A was attributed to conflict zone-related circumstances, namely poor primary hygiene practices, interrupted water supplies, and population displacement [56]. In response, the Ministry of Health reoriented public health sector towards primary care, restored disease surveillance systems and screening programmes, and initiated individual smart cards with health records and messages for required immunisations and clinic visits [56]. The importance of vaccination along with immediate water treatment and case management for the successful control of cholera outbreaks was also mentioned [56].

**Table 8:** Impact of conflict on Multiple Infectious Diseases outbreaks

*Author*	*Country, Setting, Timeframe*	*Population*	*Conflict to Disease Pathways*	*Prevention and Preparedness Strategies Suggested/Implemented*
Zhao et al., 2019 [56]	Iraq, 2003–2016	Incidence data collected from the Iraq Centre for Disease Control	• Deteriorating infrastructure during the conflict • Deterioration of water quality and sanitation • Lower vaccine coverage rate and higher vaccine failure rate • Poor primary hygiene practices • Interrupted water supplies • Population displacement	***Implemented***: The Ministry of Health: • Reoriented the public health sector towards primary care • Restored disease surveillance systems and screening programmes • Individuals carry ‘smart cards’ with their registration and health records, and receive prompts for required immunisations and clinic visits ***Suggested***: • Immediate water treatment and case management • The prevention of active disease in latently infected individuals • Federal government should manage public health security by expanding the disease surveillance system to include more types of facilities and the private sector
Haddison et al., 2020 [57]	Cameroon, Southwest Region, 2016–2018	Secondary analysis of routine surveillance data	• Reduced accessibility to health facilities due to the armed conflict • Inability to deliver drugs and supplies to health facilities as a result of attacks on the highway, blocked roads or active fighting • Abandoned health facilities due to attacks on health personnel or infrastructure • Breakdown in communication networks hampering remote supervision and data collection in facilities operating in high-risk zones • The armed conflict contributed to the internal displacement of the population and to the influx of refugees from Nigeria into Cameroon which placed an additional strain on the weakened health services in the area • The disruption of normal life and health services due to the insurgency created an enabling environment for the spread of infectious diseases.	***Implemented***: • Vaccine rollouts were made in a targeted fashion to displaced children • Engagement of community health workers in providing a continuity of care ***Suggested***: Local, national, regional, and global authorities must work together to develop risk-mitigating interventions in settings with armed conflicts to preserve the delivery and utilisation of health servicesservices.
Tarnas et al., 2021 [61]	Syria, Turkey, Lebanon, Jordan, and Iraq, 2003–2018	Population of Syria, Turkey, Lebanon, Jordan, and Iraq	• Disruption of WASH infrastructure • Mass displacement • Overcrowding in health systems that were not equipped to handle an influx of forcibly displaced people • Interruption of standard health services including routine childhood vaccination	***Implemented***: Not mentioned ***Suggested***: Not mentioned
Malik et al., 2021 [59]	Jordan, early October 2017 to January 2018	Children under-five living in Jordan	• A massive influx of Syrian refugees in Jordan placed immense pressure on the country’s over-stretched resources and affected the country’s health care system with exerted demand. • People coming through conflict-driven displacement had no or low access to healthcare and lack basic healthcare facilities. • Conflict-driven displacement has an immediate effect on child health because of access, availability and affordability issues with regard to health care services.	***Implemented***: • Given the large number of refugees in urban areas, sanitation programmes and sewage networks have been implemented among refugee concentrated camps ***Suggested***: • Concerted action is required to safeguard the health needs and avert public health emergencies due to conflict driven displacement. • Coordinated and effective measures are needed to provide the best health care services among the displaced populations to prevent health risks. • Collaborative efforts through global partners can help the countries facing the challenges of managing these health care emergencies
Haque et al., 2022 [58]	Ukraine, 24 February 2022–4 August 2022	Population of Ukraine	• Destruction of healthcare infrastructure • Bombardment of hospitals, factories and dispensaries • Destroyed roads • Delayed or interrupted vaccinations • Disruption to clean sources of water • Disruption to delivery of healthcare and health-related services • Limited access to medical care and medications during the current conflict. • In areas with active hostilities, critical supplies, including oxygen, insulin, and cancer treatments were in short supply. • Displacement of people into shelters, and overcrowded spaces with limited or no access to water and sanitation facilities	***Implemented***: Not mentioned ***Suggested***: • Ukraine’s infrastructure, health, utility and other essential systems must be rebuilt to ensure appropriate recovery for the country and its people • Continued surveillance and support are imperative to help mediate the long-term effects of the war and to rebuild Ukraine
Mobula et al., 2020 [60]	DRC, 2018	Ebola cases	• The conflict rendered certain health zones Inaccessible • Population/contact mobility • Insufficient aid for basic services impacted response activities. • Community mistrust • High population density	***Implemented for Ebola***: • Testing • Contact tracing • Isolation • Treatment • Mitigation measures (including physical distancing) ***Suggested for COVID-19***: • Application of similar infectious disease strategies and response measures as implemented for Ebola including the transfer of protocols and systems including: • Response coordination - implementation of command and control of operation centres • Surveillance systems - Creation of a monitoring framework including reporting, surveillance, contact tracing and the early detection and isolation of cases • Use of innovative data sharing platforms developed for Ebola including epidemiological support. • Risk communication and community engagement strategies • Infection prevention and control strategies • Public health emergency preparedness actions • External donor coordination

Abbreviations: WASH = water- sanitation- hygiene interventions

In the Southwest region of Cameroon, the reduction in healthcare utilisation due to reduced community accessibility to health facilities was a main consequence of the armed conflict [57]. The conflict led to the abandonment of healthcare facilities because of the attacks on health personnel and infrastructure. Moreover, there was disruption to drug deliveries to health facilities, roads were blocked, communication networks were destroyed, and the disease surveillance system was disrupted due to active fighting [57]. The disruption of normal life and health services due to the insurgency created an enabling environment for the spread of infectious diseases [57]. In response, vaccine rollouts were targeted towards displaced children and community health workers were engaged to provide a continuity of care. It was suggested that local, national, regional, and global authorities should work together to develop risk-mitigating interventions in settings with armed conflicts to preserve the delivery and utilisation of health services [57].

In Syria and neighbouring countries, the number of vector-borne disease outbreaks reported among human and animal populations increased significantly following the onset of conflict in Syria [61]. Conflict-related factors that led to the spread of infectious diseases were the disruption of WASH infrastructure and standard health services, migration, and overcrowded healthcare systems not equipped to handle a surge of forcibly displaced people [61].

In Jordan, the Syrian conflict led to the influx of Syrian refugees that placed further pressure on the country’s over-stretched resources [59]. People arriving as a result of conflict-driven displacement had no or low access to healthcare and thus were at a greater risk of exposure to diseases [59]. Additionally, conflict-driven displacement was reported to have an immediate effect on child health due to disrupting access, and limited availability and affordability of healthcare services [59]. In response, sanitation programmes and sewage networks were organised in refugee camps [59]. Coordinated and effective measures to provide the health care services among the displaced populations and collaborated efforts with global partners in managing health care emergencies were suggested [59].

In Ukraine, the Russian aggression resulted in the destruction of healthcare facilities [58]. In areas with active hostilities, critical supplies, including oxygen, insulin, and medicines were in short supply, their delivery was disrupted, and people were displaced into overcrowded shelters with limited or no access to water and sanitation facilities [58]. As a result, a reported increase in cases of HIV/AIDS, TB, and COVID-19 was observed [58]. The authors suggested that Ukraine’s infrastructure and essential systems should be rebuilt to ensure appropriate recovery for the country and its people and surveillance and support should be strengthened [58].

In DRC, the conflict rendered certain health zones inaccessible, increased population/contact mobility, reduced aid for basic services, and impacted response activities [60]. The aforementioned factors, in addition to the pre-existing community mistrust and high population density, led to increased EVD transmission [60]. The authors proposed that the strategies and response measures implemented for Ebola be used for the mitigation of COVID-19 in DRC including the transfer of protocols and systems (Table 8) [60].

## Discussion

Conflict within or between countries adversely affects population health. The studies reported here demonstrate that in conflict circumstances, affected populations are at an increased risk of infectious disease outbreaks. Infectious diseases have a significant impact on the population’s health, thus understanding the link between conflict and infectious diseases is essential. With the current systematic review, we outlined the types of infectious diseases that have been emerging in conflict-affected countries, the pathways leading from conflict to infectious disease outbreaks, and noted the prevention or response strategies and protocols implemented and recommended to prevent and control infectious disease risks.

Conflict and violence have long been associated with the introduction, transmission, and propagation of infectious disease pathogens. Overall, major overarching pathways reported in the studies identified within this review included population displacement [9–11, 15, 24, 18, 20, 27, 28, 29, 34, 33, 46, 50, 51, 56, 57, 58, 55, 62], disruption of vital infrastructure and of the healthcare system (including reduced surveillance, diagnostic delays, interrupted vaccinations [58, 61, 63], and disruption of disease control programmes) [9, 26–28, 31, 42, 43, 50, 53, 56, 57, 58], and increased population vulnerability to infection [9, 12–22, 24, 27, 32–35, 41−43, 45, 50]. Disease-specific pathways were also reported such as water contamination for cholera [17, 14, 21–23], poor injection safety [13], sexual exposure [13], risky practices for HIV [13], creation of potential mosquito-breeding sites for Dengue [53] etc. Further factors – such as the coincidence of rainy seasons, were also noted as compounding factors for vector-borne infectious diseases [46].

More specifically, infectious diseases are more likely to be introduced and transmitted during conflict situations as there are often large-scale population movements of IDPs [9–11, 15, 24, 18, 20, 27, 28, 29, 34, 33, 46, 50, 51, 56, 57, 58] and large influxes of refugees to neighbouring countries [15, 49, 57, 59, 62] which challenge resources in countries and result in overcrowded settings, poor hygienic conditions, broken infrastructure, and lack of and difficulty in receiving medical treatment [3]. These factors may also have an impact on countries not directly involved in the conflict [3]. Forcibly displaced populations are affected by a wide range of infectious disease pathogens mostly due to infections acquired in the destination country, cited as related to interrupted vaccination and a breakdown in local health infrastructure and mistrust of local medical care [63]. These findings are consistent with prior studies that have demonstrated low risks of imported acute infectious diseases impacting host country epidemiology while crowding associated with temporary resettlement increases the risk of outbreaks among displaced residents [63]. More specifically, conflicts and wars led to forced large-scale population migration, large numbers of IPDs, and influx of refugees which in turn led to high population density and large numbers of people that moved into overcrowded shelter arrangements with limited access to sanitation facilities, safe water and limited or no access to medical care. Another factor related to the introduction and transmission of infectious diseases was the mass movement of people between IDP camps and host communities [52]. Additionally, internal population displacement was associated with the geographic spread of infectious diseases from the regions involved in the armed conflict to the rest of the country and to neighbouring countries (such as between Ukraine and Poland) [64] with phylodynamic evidence supporting this case [65, 66]. High population density during evacuation plans [27] and the lack of social distancing among militaries and prisoners [43, 67], were also reported as pathways leading to the transmission of infectious diseases.

Conflicts have been noted as a direct cause of the destruction of electricity, water, transportation, and health infrastructure as well as the disruption in functioning health systems [27, 28, 31, 42, 43, 50, 53, 56–58]. Organised attacks on power, water, and transportation infrastructure were reported across studies. In areas with active hostilities, roads were blocked and destroyed, highways were attacked, communication networks were broken [57, 58], power failures/loss of electrical power occurred [16, 31], clean water sources were disrupted, and critical medical products, including vaccines, were in short supply with their deliveries to health facilities disrupted [27, 29, 57, 58, 60]. Furthermore, organised attacks on health infrastructure, including the bombing of hospitals, factories and dispensaries as well as the targeting and persecution of healthcare personnel resulted in the destruction and abandonment of healthcare facilities [39, 40, 43, 57], a significant reduction in medical personnel, and ultimately the disruption of disease surveillance systems [9, 26, 56, 57] and of the necessary healthcare services. Surveillance systems are often weak in conflict situations, resulting in delays in the detection and reporting of epidemics [37, 40]. Armed conflicts also were reported to have caused treatment interruptions [9–11, 34, 35, 46], limited patient-provider consultations [57] and limited follow-up [40], especially for people in areas with active hostilities. Other factors that aided transmission of water-borne infectious diseases were reported and these included disruptions in sewage management and wastewater treatment facilities as well as inactive pipe water distribution systems that resulted in severe shortages of clean water [16, 17, 56].

Additionally, certain war zones were rendered inaccessible to governmental organisations and NGOs due to active fighting or because they were controlled by anti-government organisations. In these areas, the population could not be reached, medicine could not be delivered or delivered in time, administration of vaccinations was interrupted [58, 61, 63], and access to treatment was limited [34, 35, 60]. The lack of vaccination plans in active conflict areas was also noted [27].

Finally, conflicts may fuel epidemics by increasing population vulnerabilities due to poverty, malnutrition, medical deprivation, uncertainty, and a breakdown of social structures. Shortages of food, drinking water, and medication were important conflict-to-disease-related factors in the studied literature. Conflicts make populations vulnerable and insecure with limited access to safe water, food, healthcare, and other basic necessities [9, 13–20, 24, 27, 37, 42, 43, 56, 58]. Moreover, migrants are often emotionally and physically stressed and thus have low immunity to disease endemics in the new area [35]. Children are the most vulnerable group as malnutrition, lack of basic necessities, and limited access to healthcare make them more susceptible to infectious diseases if neither vaccinated nor previously exposed [45, 52, 59]. In addition, increasing community distrust of the government and the public health response was reported in some of the affected countries [38–40, 42, 44, 60]. Public mistrust in the governmental and international organisations that run vaccination campaigns resulted in increased rates of unvaccinated adults and children with low immunity to vaccine-preventable diseases [44], impeding the control of vaccine-preventable infectious disease outbreaks. Finally, refugees and vulnerable people due to conflicts were reported to be affected by sexual violence and abuse, increased drug use, lack of health infrastructure, education, income, and basic needs, and social structure breakdown [68].

On the antipode, prevention strategies, preparedness plans, and emergency response procedures are the key to effective epidemic control. The most important public health detection and prevention strategies reported in the studied literature included: disease awareness programmes [9, 13, 17–19, 26, 28, 31, 49, 50] and the education of the general population [24], implementation of WASH strategies [15, 18, 20, 24], organisation of vaccination sites and vaccination campaigns [10, 37–39, 41, 42, 44, 51, 52], access to healthcare and applying preventable services in conflict zones/camps, training and recruiting healthcare workers [26, 30, 32, 33, 43, 49], developing surveillance and response protocols and ensuring cooperation/coordination between international agencies/nongovernmental organisations, national and regional authorities, and local/front-line NGOs [30, 32, 38–40, 44, 49, 52, 57, 59].

Disease education/awareness programmes and community mobilisation/engagement campaigns were considered a necessary part of any plan to successfully prevent epidemics for the majority of the studied infectious diseases [9, 10, 18, 26, 28, 31, 49, 50] Measures included education and sensitising programmes for infectious diseases targeting at-risk populations and populations in host communities [9] through culturally appropriate information on symptoms and how to seek medical advice and treatment [49]. Disease awareness programmes for civilians [10, 28, 31], and importantly for military personnel [31] on personal protective measures, immunisations, chemoprophylaxis and surveillance were suggested. The distribution of public awareness material on proper personal hygiene, food, and water safety [19] for other infectious diseases was also suggested. In the case of HIV infection, the emphasis was placed on educating young people aged 15 to 24 [9] with the development of educational/awareness materials in appropriate languages [68], programmes for in-school and out-of-school youth [68], peer education [68], youth centres [68], sports/drama groups [68], and programmes aimed at reducing teenage pregnancy and sexual violence [68].

Furthermore, investing and enabling health care and preventative services in locations that have been disrupted by conflict and in locations with displaced populations was stressed across the studied literature. The reconstruction and rehabilitation of health centres and hospitals [9, 24] within conflict areas have been noted as a primary response strategy. Infection control procedures should also be instituted in healthcare centers including the development of procedures in establishing an isolation facility, ensuring safe water, sanitation and waste disposal and providing personal protective equipment for staff within hospitals [2]. The provision of therapeutic and diagnostic supplies and testing equipment [28] and preparing with medications, and equipment standard medical facilities with intensive care capacity [31] have also been noted as key response measures, including the importance and need to adapt strategies, guidelines and tools [26], such as treatment regimens [32] and the expansion of user-friendly short-course DOTS for TB [32] in conflict settings.

The recruitment of health staff and the establishment of early disease detection training programmes for healthcare professionals are also critical measures to maintaining healthcare in conflict situations and in post-conflict rehabilitation [27, 28, 32, 33, 43, 49]. Similarly, improved preparedness of the public health authorities for surveillance (including public health laboratories at central and regional levels) and response systems [19] is also essential.

For the vaccine preventable diseases identified within the context of this review, the studies suggested the implementation of several approaches towards vaccination strategies [13, 27, 37, 39, 41, 44, 51], aimed to cover at-risk populations both within combat areas and amongst displaced populations with vaccination at entry and exit screenings [39]. Studies also described numerous approaches towards implementing vaccination of at-risk populations such as displaced persons, including organising vaccination campaigns at fixed posts where the team was based at the health facilities and at temporary post located at strategic areas such as transit points, bus stations and refuge sites [29, 51, 52], as well as the use of mobile outreach teams [51]. In addition, the creation and use of medical supplies and global vaccine stockpiles were suggested as mitigation measures that could be available for rapid deployment in emergency and outbreak situations [16, 19]. In some cases, this is easily achievable, as with the provision of OCVs [15, 20, 24] while in other circumstances protocols including the maintenance of a cold chain for transport and the use of trained teams are needed [39]. In all cases, encouraging the community to participate at every level of the vaccine rollout improves public trust which is essential for the effective implementation of vaccine rollout plans [29, 38–40].

The importance of collaborative efforts between international agencies and nongovernmental organisations, national and regional authorities and local/front-line NGOs was stressed in most of the studied articles [30, 38–40, 43, 44, 49, 51, 57, 59]. During active conflicts, interoperability and biosurveillance information sharing across agencies are needed [30]. In order to maintain the delivery and utilisation of health care in areas affected by armed conflicts, it was advised that local, national, regional, and international authorities cooperate [57]. It was further recommended that international financial and logistical support should be given to front-line NGOs to reach and safely provide healthcare to isolated/hard-to-reach populations [43]. Also, international organisations providing health services should be given unconditional safe access to conflict zones. Finally, political commitment is necessary to achieve as far as possible uninterrupted and safe medical and humanitarian aid to conflict zones [32, 44], as well as support for post-conflict recovery.

Extended wars and conflicts often make people reliant on foreign aid. This need for international aid often lasts for a long time even after peace is restored. Therefore multi-agency humanitarian assistance must promote effective, efficient, and socio-culturally suitable healthcare in a sustainable way [69]. It is of critical importance to improve the quality of life to avoid and minimise disease occurrence among both displaced and conflict-affected population’s [70]. As such, international organisations that provide health and humanitarian services should be given unconditional access to conflict zones [32] and should be allowed to function impartially within conflict [43]. The government and opposition groups must be engaged with to ease passage of aid and access to conflict-affected populations [71].

Last but not least, studies have noted the importance of a proactive identification of vulnerabilities and locations for interventions through surveillance [14, 19, 31, 38, 43, 46, 50, 51, 58, 62]. It is essential to improve the preparedness of the public health authorities by increasing international surveillance [14, 19, 31, 38, 43, 46, 50, 51, 58, 62] and international financial and logistical support for vaccine and immunisation of the population, especially in conflict-torn countries [43]. Improved methods for measuring population movement within and between countries during complex emergencies are needed [20], through multisource surveillance techniques [20] and through the development of predictive tools to identify vulnerabilities and settings posing a high a risk of infectious disease [16]. While advanced surveillance systems (i.e. genomics) are important, easy applicable early warning systems, such as tally sheets to monitor campaign coverage [51], are also beneficial. To this extent, tracking spatiotemporal patterns of populations and disease transmission [14], along with the early detection of outbreaks supporting the prediction of areas at increased risk for infectious disease introductions/outbreaks is essential. Surveillance and broad testing policies, not only for severe hospitalised cases but also for milder, asymptomatic cases are necessary response measures that could contribute to lower mortality [30]. Furthermore, the creation of effective and targeted public health emergency response plans based on the acquired information is also needed to enable timely and effective mitigation of future health challenges arising in conflict-affected countries and in countries hosting displaced populations.

### Strengths and limitations

The systematic investigation of the literature, the thorough assessment, data extraction, quality appraisal, and synthesis of published evidence are strengths of the current review. However, it is important to acknowledge certain limitations. This review focused on peer-reviewed evidence available in the English language. Therefore, relevant information published in other languages may be missing. Additionally, our inclusion criteria did not impose geographical restrictions, necessitating cautious interpretation of the findings in light of documented variations across countries and different disease outbreaks. Furthermore, while this review elucidates several factors contributing to infectious disease outbreaks in conflict settings, it is conceivable that not all causal pathways linking conflicts and infectious diseases were identified, as monitoring and assessing all pathways within conflict zones can pose significant challenges. Hence, future research endeavours should aim to shed further light on additional disease-specific pathways that precipitate outbreaks. The identification of qualitative information from key informants and/or patients within conflict zones could enhance our understanding of the issue. Future studies should consider addressing this aspect. In addition, incorporating econometric analyses would be interesting to indicate the impact of conflict on infectious diseases in economic terms.

## Conclusion

Conflicts play a direct and indirect role in the transmission and propagation of infectious diseases due to population displacement, overcrowded settlements with poor sanitary conditions, disruption of infrastructure, reduction in functioning healthcare system, shortages of food, limited access to clean drinking water, medications and healthcare, delays in diagnosis and disruptions in vaccine coverage. The most important prevention and preparedness strategies for an infectious disease outbreak in a conflict situation included education/awareness campaigns, reconstruction of healthcare facilities including recruitment and training of healthcare workers, strengthening surveillance and early warning systems, enabling access to healthcare in conflict zones, deployment of global vaccine and medical stockpiles, and implementation of water- sanitation- hygiene interventions. Finally, collaboration between humanitarian and health actors, community engagement, and political will were identified as critical factors in responding to infectious disease outbreaks in conflict settings. Through identifying commonly reported risk pathways as well as mitigation strategies, the findings of this review may assist decision-makers to implement evidence-based preparedness and response strategies for the timely and effective mitigation of future infectious disease outbreaks in conflict areas.

### Electronic supplementary material

Below is the link to the electronic supplementary material.

Supplementary Material 1

## Data Availability

Not applicable.
