# Supplementary material for: The impact of conflict on infectious disease: a systematic literature review
Source: Confl Health. 2024 Apr 8;18:27. doi: 10.1186/s13031-023-00568-z (PMC11000310; doi:10.1186/s13031-023-00568-z)
Supplement: Supplementary file 1 — Supplementary Material 1 [file 13031_2023_568_MOESM1_ESM.docx]

Appendix

**Supplement to: *The impact of conflict on infectious disease: A systematic literature review***

[Supplementary Table 1 – PRISMA Checklist (prisma-statement.org) 2](#_Toc150427655)

[Supplementary Table 2 – inclusion and exclusion criteria 4](#_Toc150427656)

[Supplementary Table 3 – Search Strategy 5](#_Toc150427657)

[Supplementary Table 4 – Quality appraisal 9](#_Toc150427658)

[Cross-sectional studies 9](#_Toc150427659)

[Opinion papers 10](#_Toc150427660)

[Quasi-experimental studies 11](#_Toc150427661)

[Case series 12](#_Toc150427662)

[Cohort studies 12](#_Toc150427663)

[Case report 13](#_Toc150427664)

[Prevalence studies 13](#_Toc150427665)

[References 13](#_Toc150427666)

# Supplementary Table 1 – PRISMA Checklist (prisma-statement.org)

Our systematic review adhered to the PRISMA statement or Preferred Reporting Items for Systematic Reviews and Meta-Analyses. The PRISMA statement provides a checklist^1^ for review authors on how to report a systematic review, presented in **Supplementary Table 1.**

| **Section and Topic** | **Item #** | **Checklist item** | **Location where item is reported** |
| --- | --- | --- | --- |
| **TITLE** | | |  |
| Title | 1 | Identify the report as a systematic review. | p. 1 |
| **ABSTRACT** | | |  |
| Abstract | 2 | See the PRISMA 2020 for Abstracts checklist. | p. 2 |
| **INTRODUCTION** | | |  |
| Rationale | 3 | Describe the rationale for the review in the context of existing knowledge. | p. 4 |
| Objectives | 4 | Provide an explicit statement of the objective(s) or question(s) the review addresses. | p. 4 |
| **METHODS** | | |  |
| Eligibility criteria | 5 | Specify the inclusion and exclusion criteria for the review and how studies were grouped for the syntheses. | p. 5 |
| Information sources | 6 | Specify all databases, registers, websites, organisations, reference lists and other sources searched or consulted to identify studies. Specify the date when each source was last searched or consulted. | p. 5, Supplementary table 2 |
| Search strategy | 7 | Present the full search strategies for all databases, registers and websites, including any filters and limits used. | p. 5, Supplementary table 2 |
| Selection process | 8 | Specify the methods used to decide whether a study met the inclusion criteria of the review, including how many reviewers screened each record and each report retrieved, whether they worked independently, and if applicable, details of automation tools used in the process. | p. 5 |
| Data collection process | 9 | Specify the methods used to collect data from reports, including how many reviewers collected data from each report, whether they worked independently, any processes for obtaining or confirming data from study investigators, and if applicable, details of automation tools used in the process. | p. 5 |
| Data items | 10a | List and define all outcomes for which data were sought. Specify whether all results that were compatible with each outcome domain in each study were sought (e.g. for all measures, time points, analyses), and if not, the methods used to decide which results to collect. | p. 5 |
|  | 10b | List and define all other variables for which data were sought (e.g. participant and intervention characteristics, funding sources). Describe any assumptions made about any missing or unclear information. | p. 5 |
| Study risk of bias assessment | 11 | Specify the methods used to assess risk of bias in the included studies, including details of the tool(s) used, how many reviewers assessed each study and whether they worked independently, and if applicable, details of automation tools used in the process. | p. 5 |
| Effect measures | 12 | Specify for each outcome the effect measure(s) (e.g. risk ratio, mean difference) used in the synthesis or presentation of results. | p. 5 |
| Synthesis methods | 13a | Describe the processes used to decide which studies were eligible for each synthesis (e.g. tabulating the study intervention characteristics and comparing against the planned groups for each synthesis (item #5)). | p. 5 |
|  | 13b | Describe any methods required to prepare the data for presentation or synthesis, such as handling of missing summary statistics, or data conversions. | p. 5 |
|  | 13c | Describe any methods used to tabulate or visually display results of individual studies and syntheses. | p. 5 |
|  | 13d | Describe any methods used to synthesize results and provide a rationale for the choice(s). If meta-analysis was performed, describe the model(s), method(s) to identify the presence and extent of statistical heterogeneity, and software package(s) used. | Not relevant |
|  | 13e | Describe any methods used to explore possible causes of heterogeneity among study results (e.g. subgroup analysis, meta-regression). | Not relevant |
|  | 13f | Describe any sensitivity analyses conducted to assess robustness of the synthesized results. | Not relevant |
| Reporting bias assessment | 14 | Describe any methods used to assess risk of bias due to missing results in a synthesis (arising from reporting biases). | p. 5 |
| Certainty assessment | 15 | Describe any methods used to assess certainty (or confidence) in the body of evidence for an outcome. | Not relevant |
| **RESULTS** | | |  |
| Study selection | 16a | Describe the results of the search and selection process, from the number of records identified in the search to the number of studies included in the review, ideally using a flow diagram. | p. 6 |
|  | 16b | Cite studies that might appear to meet the inclusion criteria, but which were excluded, and explain why they were excluded. | p. 6 |
| Study characteristics | 17 | Cite each included study and present its characteristics. | p. 6-37 |
| Risk of bias in studies | 18 | Present assessments of risk of bias for each included study. | Supplementary Table 3 |
| Results of individual studies | 19 | For all outcomes, present, for each study: (a) summary statistics for each group (where appropriate) and (b) an effect estimate and its precision (e.g. confidence/credible interval), ideally using structured tables or plots. | p. 6-37 |
| Results of syntheses | 20a | For each synthesis, briefly summarise the characteristics and risk of bias among contributing studies. | Supplementary Table 3 |
|  | 20b | Present results of all statistical syntheses conducted. If meta-analysis was done, present for each the summary estimate and its precision (e.g. confidence/credible interval) and measures of statistical heterogeneity. If comparing groups, describe the direction of the effect. | Not relevant |
|  | 20c | Present results of all investigations of possible causes of heterogeneity among study results. | Not relevant |
|  | 20d | Present results of all sensitivity analyses conducted to assess the robustness of the synthesized results. | Not relevant |
| Reporting biases | 21 | Present assessments of risk of bias due to missing results (arising from reporting biases) for each synthesis assessed. | Supplementary Table 3 |
| Certainty of evidence | 22 | Present assessments of certainty (or confidence) in the body of evidence for each outcome assessed. | Supplementary Table 3 |
| **DISCUSSION** | | |  |
| Discussion | 23a | Provide a general interpretation of the results in the context of other evidence. | p. 37-42 |
|  | 23b | Discuss any limitations of the evidence included in the review. | p. 43 |
|  | 23c | Discuss any limitations of the review processes used. | p. 43 |
|  | 23d | Discuss implications of the results for practice, policy, and future research. | p. 43 |
| **OTHER INFORMATION** | | |  |
| Registration and protocol | 24a | Provide registration information for the review, including register name and registration number, or state that the review was not registered. | Not registered |
|  | 24b | Indicate where the review protocol can be accessed, or state that a protocol was not prepared. | p. 5 |
|  | 24c | Describe and explain any amendments to information provided at registration or in the protocol. | Not relevant |
| Support | 25 | Describe sources of financial or non-financial support for the review, and the role of the funders or sponsors in the review. | Funding |
| Competing interests | 26 | Declare any competing interests of review authors. | Declaration of interests |
| Availability of data, code and other materials | 27 | Report which of the following are publicly available and where they can be found: template data collection forms; data extracted from included studies; data used for all analyses; analytic code; any other materials used in the review. | Data sharing |

*From:* Page MJ, McKenzie JE, Bossuyt PM, Boutron I, Hoffmann TC, Mulrow CD, et al. The PRISMA 2020 statement: an updated guideline for reporting systematic reviews. *BMJ* 2021;**372:n71**. doi: 10.1136/bmj.n71. For more information, visit: http://www.prisma-statement.org/

# Supplementary Table 2 – inclusion and exclusion criteria

|  | Inclusion | Exclusion |
| --- | --- | --- |
| Population | Any population | None |
| Geographical region | Any geographical region | None |
| Data/ Outcomes | Any data/ outcome related to infectious diseases in  a conflict setting |  |
| Study Design | Any study design, including field reports and perspective articles | None |
| Language | English language only |  |
| Timespan | January 2000 to October 2023 |  |

# Supplementary Table 3 – Search Strategy

The search terms used for each database search are presented in **Supplementary Table 3**. Subject heading terms and free text words were used to develop a comprehensive search strategy (JLB). Studies that met the search criteria were evaluated for their validity and reliability. Systematic and non-systematic literature reviews were excluded, but their references were screened. Initially, a pilot round of title/abstract screening was conducted, where a random sample of 100 titles was screened for eligibility independently by two reviewers (ZP, KA) to enable consistency in screening and to identify areas for amendments in the inclusion criteria. A high measure of inter-rater agreement was achieved (percentage agreement >90%), hence the remaining titles were distributed to be screened independently by two reviewers. For the full-text screening, all full texts were screened for eligibility independently by two reviewers (KN, KA). Any disagreements were discussed with a third reviewer (CV). Documents that passed the inclusion criteria on the full-text screening were included in the review.

| **Database: Ovid MEDLINE(R) ALL <1946 to October 24, 2023>** |  |
| --- | --- |
| **KEY TERMS** | **HITS** |
| 1 (disease outbreak or outbreak or epidemic or pandemic or "public health emergency").mp. or exp communicable diseases/ or exp disease outbreak/ | 997406 |
| 2 (avian flu or ebola or EVD or H1N1 or H5N1 or infectious disease or influenza or swine flu or flu or MERS or Middle East Respiratory Syndrome).mp. | 233632 |
| 3 (SARS or Severe Acute Respiratory syndrome or measles or zika or cholera or H7N9 or dengue or fever or plague or fever or malaria or polio).mp. | 674510 |
| 4 (Bacillus cereus or Campylobacter jejuni or Clostridium or Cryptosporidium or Cyclospora cayetanensis or (E adj coli) or Hepatitis A or Listeria monocytogenes or Noroviruses or Salmonella or Shigella or Staphylococcus aureus or Staphylococcus or Vibrio parahaemolyticus or Vibrio vulnificus).mp. | 589234 |
| 5 (Diphtheria or Haemophilus influenzae type b or Hib or Hepatitis B or Human Papillomavirus or HPV).mp. | 199721 |
| 6 ((Meningococcal adj Infection$) or Mump$ or Pertussis or Whooping Cough or Pneumococcal Infection$ or Polio or Rotavirus or Rubella or German Measles or Tetanus or varicella or chicken pox or vectorbourne diseases or vector?bourne disease$ or waterbourne diseases or water?bourne disease$ or Cholera or Diarrhoea or diarrhoea).mp. | 296493 |
| 7 (Typhoid fever or Giardiasis or Schistosomiasis or Dracunculiasis or Dysentery or Cryptosporidiosis or amoebiasis or Traveler$s diarrhea or travelers diarrhoea).mp. | 76719 |
| 8 exp infectious disease medicine/ or exp malaria/ or exp influenza, human/ or SARS virus/ or exp norovirus/ or exp coronavirus infections/ or exp measles/ or exp poliomyelitis/ or exp chickenpox/ | 435322 |
| 9 (anthrax or botulism or brucellosis or campylobacter enteritis or chikungunya or chlamydia$ or CJD or Creutzfeldt?Jakob).mp. | 73741 |
| 10 (diptheria or echinococcosis or gonococcal or haemophilus influenzae or hepatitis or HIV or AIDS or human immunodeficiency virus or acquired immunodeficiency syndrome).mp. | 830590 |
| 11 (legionnaires?disease or leptospirosis or listeriosis or lyme or streptococcus pneumoniae or Q fever or rabies or congenital rubella or salmonella or shiga toxin or verocytotoxin?producing E?coli or STEC or VTEC or HUS or haemoltic?uraemic or hemoltic?uremic).mp. | 210036 |
| 12 (shigellosis or smallpox or syphilis or congenital syphilis or tick?borne viral encephalitis or congenital toxoplasmosis or trichinellosis or tuberculosis or TB or typhoid or paratyphoid or VHF or viral hemorrhagic fever$ or viral haemorrhagic fever$ or West Nile virus or Yellow fever or (enteritis adj3 yersinia)).mp. | 395889 |
| 13 exp coronavirus/ or exp Coronavirus disease 2019/ or exp coronavirus infections/ or Coronaviridae Infections/ or Coronaviridae/ or SARS-CoV-2/ or COVID-19/ or (("2019" adj (novel or new) adj corona*) or ("2019" adj (CoV or nCoV)) or (coronavirus adj (disease adj "2019")) or COVID19 or COVID-19 or ((Novel or New) adj Corona*) or SARS2 or SARS-CoV-2 or (SARS adj2 (coronaviridae or coronavirus)) or ((sars or Coronavirus) adj "2") or nCov or 2019ncov or (severe adj acute adj respiratory adj syndrome) or SARs or Sars-cov or ((sars-associated or sars-related) adj (cov or coronavirus))).mp. or Betacoronavirus 1/ or Betacoronavirus/ | 411394 |
| 14 or/1-13 | 3188881 |
| 15 (insurgenc$ or "mass atrocit$" or "human rights violation$" or "crimes against humanity" or "mass casualt$" or "mass fatalit$").mp. | 5823 |
| 16 (infrastructure or immuni?ation program$ or migration or displacement or humanitarian assistance or overcrowding or sanitary conditions or sanitation or population vulnerability or late detection or late warning or environmental impact$ or sanitation or sewers or housing or displacement or movement or crowding or drought or aid workers or access or compliance).mp. | 1675108 |
| 17 (public service$ or loss of service$ or stabilisation or recovery or secondary effects or surveillance or disease control or insecurit$ or resource or coordination or temporary settlement$ or safe water or disease vector$ or trained staff or under?nutrition or mal?nutrition or vaccine or vaccination or investment or education or infection control).mp. | 2853740 |
| 18 15 or 16 or 17 | 4288494 |
| 19 "Warfare and Armed Conflicts"/ | 42 |
| 20 Armed Conflicts/ or Warfare/ or Biological Warfare/ or Bioterrorism/ or Chemical Warfare/ or Chemical Terrorism/ or Nuclear Warfare/ or Psychological Warfare/ or War Crimes/ or Ethnic Cleansing/ or Genocide/ or Holocaust/ or War Exposure/ or War-Related Injuries/ or (DNBI or "disease and non?battle injur$").mp. | 38787 |
| 21 armed conflicts/ or afghan campaign 2001-/ or gulf war/ or iraq war, 2003-2011/ or september 11 terrorist attacks/ or ((Afghanistan or Bosnia or Kosovo or Yugoslav$ or Ukraine) adj2 (war$ or conflict$ or attack$ or violence)).mp. 7959 | 8525 |
| 22 (armed conflict or armed conflicts or gulf war or iraq war or war time or wartime or afghan war or army to armed forces or troops or armed service or ((armed or zone or political or civil) adj3 (conflict or conflicts or attack or attacks or war or wars or no fly or violence))).mp. | 17425 |
| 23 (war related injuries or war related traumas or war related injury or war related trauma or (militant group or militant groups or militant organization or militant organizations or militant organisation or militant organisations) or (biological terrorism or bioterrorism or biowarfare or chemical terrorism or ethnic cleansing or ethnic cleansings or gas poisoning or genocide or holocaust or holocausts or nuclear terrorism or war exposure or war exposures)).mp. | 11528 |
| 24 19 or 20 or 21 or 22 or 23 | 57859 |
| 25 14 and 18 and 24 | 4063 |
| 26 limit 25 to yr="1990 - Current" | 3907 |

| **Database: EMBASE <1946 to October 23, 2023>** |  |
| --- | --- |
| **KEY TERMS** | **HITS** |
| 1 (disease outbreak or outbreak or epidemic or pandemic or "public health emergency").mp. or exp communicable diseases/ or exp disease outbreak/ | 642776 |
| 2 (avian flu or ebola or EVD or H1N1 or H5N1 or infectious disease or influenza or swine flu or flu or MERS or Middle East Respiratory Syndrome).mp. | 291108 |
| 3 (SARS or Severe Acute Respiratory syndrome or measles or zika or cholera or H7N9 or dengue or fever or plague or fever or malaria or polio).mp. | 916126 |
| 4 (Bacillus cereus or Campylobacter jejuni or Clostridium or Cryptosporidium or Cyclospora cayetanensis or (E adj coli) or Hepatitis A or Listeria monocytogenes or Noroviruses or Salmonella or Shigella or Staphylococcus aureus or Staphylococcus or Vibrio parahaemolyticus or Vibrio vulnificus).mp. | 732839 |
| 5 (Diphtheria or Haemophilus influenzae type b or Hib or Hepatitis B or Human Papillomavirus or HPV).mp. | 304703 |
| 6 ((Meningococcal adj Infection$) or Mump$ or Pertussis or Whooping Cough or Pneumococcal Infection$ or Polio or Rotavirus or Rubella or German Measles or Tetanus or varicella or chicken pox or vectorbourne diseases or vector?bourne disease$ or waterbourne diseases or water?bourne disease$ or Cholera or Diarrhea or diarrhoea).mp. | 532217 |
| 7 (Typhoid fever or Giardiasis or Schistosomiasis or Dracunculiasis or Dysentery or Cryptosporidiosis or amoebiasis or Traveler$s diarrhea or travelers diarrhoea).mp. | 67093 |
| 8 exp infectious disease medicine/ or exp malaria/ or exp influenza, human/ or SARS virus/ or exp norovirus/ or exp coronavirus infections/ or exp measles/ or exp poliomyelitis/ or exp chickenpox/ | 629737 |
| 9 (anthrax or botulism or brucellosis or campylobacter enteritis or chikungunya or chlamydia$ or CJD or Creutzfeldt?Jakob).mp. | 95828 |
| 10 (diptheria or echinococcosis or gonococcal or haemophilus influenzae or hepatitis or HIV or AIDS or human immunodeficiency virus or acquired immunodeficiency syndrome).mp. | 1141622 |
| 11 (legionnaires?disease or leptospirosis or listeriosis or lyme or streptococcus pneumoniae or Q fever or rabies or congenital rubella or salmonella or shiga toxin or verocytotoxin?producing E?coli or STEC or VTEC or HUS or haemoltic?uraemic or hemoltic?uremic).mp. | 249354 |
| 12 (shigellosis or smallpox or syphilis or congenital syphilis or tick?borne viral encephalitis or congenital toxoplasmosis or trichinellosis or tuberculosis or TB or typhoid or paratyphoid or VHF or viral hemorrhagic fever$ or viral haemorrhagic fever$ or West Nile virus or Yellow fever or (enteritis adj3 yersinia)).mp. | 394630 |
| 13 exp coronavirus/ or exp Coronavirus disease 2019/ or exp coronavirus infections/ or Coronaviridae Infections/ or Coronaviridae/ or SARS-CoV-2/ or COVID-19/ or (("2019" adj (novel or new) adj corona*) or ("2019" adj (CoV or nCoV)) or (coronavirus adj (disease adj "2019")) or COVID19 or COVID-19 or ((Novel or New) adj Corona*) or SARS2 or SARS-CoV-2 or (SARS adj2 (coronaviridae or coronavirus)) or ((sars or Coronavirus) adj "2") or nCov or 2019ncov or (severe adj acute adj respiratory adj syndrome) or SARs or Sars-cov or ((sars-associated or sars-related) adj (cov or coronavirus))).mp. or Betacoronavirus 1/ or Betacoronavirus/ | 489422 |
| 14 or/1-13 | 4000989 |
| 15 (insurgenc$ or "mass atrocit$" or "human rights violation$" or "crimes against humanity" or "mass casualt$" or "mass fatalit$").mp. | 5296 |
| 16 (infrastructure or immuni?ation program$ or migration or displacement or humanitarian assistance or overcrowding or sanitary conditions or sanitation or population vulnerability or late detection or late warning or environmental impact$ or sanitation or sewers or housing or displacement or movement or crowding or drought or aid workers or access or compliance).mp. | 2424454 |
| 17 (public service$ or loss of service$ or stabilisation or recovery or secondary effects or surveillance or disease control or insecurit$ or resource or coordination or temporary settlement$ or safe water or disease vector$ or trained staff or under?nutrition or mal?nutrition or vaccine or vaccination or investment or education or infection control).mp. | 3812388 |
| 18 15 or 16 or 17 | 5846818 |
| 19 "Warfare and Armed Conflicts"/ | 1206 |
| 20 Armed Conflicts/ or Warfare/ or Biological Warfare/ or Bioterrorism/ or Chemical Warfare/ or Chemical Terrorism/ or Nuclear Warfare/ or Psychological Warfare/ or War Crimes/ or Ethnic Cleansing/ or Genocide/ or Holocaust/ or War Exposure/ or War-Related Injuries/ or (DNBI or "disease and non?battle injur$").mp. | 49346 |
| 21 armed conflicts/ or afghan campaign 2001-/ or gulf war/ or iraq war, 2003-2011/ or september 11 terrorist attacks/ or ((Afghanistan or Bosnia or Kosovo or Yugoslav$ or Ukraine) adj2 (war$ or conflict$ or attack$ or violence)).mp. 7959 | 42087 |
| 22 (armed conflict or armed conflicts or gulf war or iraq war or war time or wartime or afghan war or army to armed forces or troops or armed service or ((armed or zone or political or civil) adj3 (conflict or conflicts or attack or attacks or war or wars or no fly or violence))).mp. | 13803 |
| 23 (war related injuries or war related traumas or war related injury or war related trauma or (militant group or militant groups or militant organization or militant organizations or militant organisation or militant organisations) or (biological terrorism or bioterrorism or biowarfare or chemical terrorism or ethnic cleansing or ethnic cleansings or gas poisoning or genocide or holocaust or holocausts or nuclear terrorism or war exposure or war exposures)).mp. | 9492 |
| 24 19 or 20 or 21 or 22 or 23 | 70442 |
| 25 14 and 18 and 24 | 6511 |
| 26 limit 25 to yr="1990 - Current" | 6400 |

# Supplementary Table 4 – Quality appraisal

The methodological quality (or risk of bias) of the included studies was evaluated using the Joanna Briggs Institute (JBI) standardised critical appraisal tools.^2^ We encountered a couple of issues while deciding the appropriate tool for some of the studies, as there are many distinct study designs in the literature some of which cannot fit to a particular JBI’s tool category. In particular, the ecological/correlation study by Vasylyeva et al., 2018^3^ is a descriptive study and as such none of the JBI tools is appropriate. However, people have used the JBI analytical cross-sectional study tool for such studies, which then score very low. Moreover, for the time-series study of Sedda et al., 2015^4^ there no specific JBI tool. The same applies to the study of Simpson et al., 2022^5^ as a time series model there isn't a tool for this study. Similarly, there is no specific JBI tool for the time series study of Wells et al., 2019.^6^ Additionally, both the pre-post studies of Tarnas et al., 2021^7^ and of Haddison et al., 2020^8^ are observational, without an application of an intervention and as such no JBI tool is appropriate. Finally, Mobula et al., 2020^9^ is described as a case series for Ebola outbreak but is in fact a qualitative study. As such we chose assess this study with the checklist for text and opinion tool.

### Cross-sectional studies

Quality Appraisal for the included cross-sectional studies (n=17).

Answers: Yes, No, Unclear or Not/Applicable.

Every “Yes” or “Not Applicable (N/A)” gives a score. 0-4 scores=low quality. 5-8 scores=intermediate quality, and 9-12 scores=high quality.

| Study | Were the criteria for inclusion in the sample clearly defined? | Were the study subjects and the setting described in detail? | Was the exposure measured in a valid and reliable way? | Were objective, standard criteria used for measurement of the condition? | Were confounding factors identified? | Were strategies to deal with confounding factors stated? | Were the outcomes measured in a valid and reliable way? | Was appropriate statistical analysis used? | Rating |
| --- | --- | --- | --- | --- | --- | --- | --- | --- | --- |
| Akil and Ahmad, 2016^10^ | YES | YES | YES | YES | NO | NO | YES | YES | 6/8 |
| Alawieh et al., 2014^11^ | YES | YES | YES | YES | YES | YES | YES | N/A | 8/8 |
| Alghazali et al., 2019^12^ | YES | YES | YES | YES | NO | NO | YES | N/A | 6/8 |
| Ali et al., 2012^13^ | YES | YES | YES | YES | YES | YES | YES | N/A | 8/8 |
| Altmann et al., 2017^14^ | YES | YES | NO | NO | NO | NO | YES | N/A | 4/8 |
| Betsi et al., 2006^15^ | YES | YES | NO | YES | NO | NO | YES | N/A | 5/8 |
| Boyong et al., 2018^16^ | YES | YES | YES | YES | NO | NO | YES | YES | 6/8 |
| CDC 2004^17^ | YES | YES | YES | YES | NO | NO | N/A | N/A | 6/8 |
| Daw et al., 2022^18^ | YES | YES | YES | YES | NO | NO | YES | YES | 6/8 |
| Dureab et al., 2018^19^ | YES | YES | YES | YES | NO | NO | YES | YES | 6/8 |
| Gele and Bjune, 2010^20^ | YES | YES | NO | YES | NO | NO | YES | YES | 6/8 |
| Haque et al., 2022^21^ | YES | YES | YES | YES | NO | NO | YES | N/A | 6/8 |
| Malik et al., 2021^22^ | YES | YES | YES | YES | YES | YES | YES | YES | 8/8 |
| Njoh et al., 2022^23^ | YES | YES | YES | YES | YES | YES | YES | YES | 8/8 |
| Vasylyeva et al., 2018^3^ | YES | YES | YES | YES | YES | YES | YES | YES | 8/8 |
| Weil et al., 2021^24^ | YES | YES | YES | YES | YES | YES | YES | YES | 8/8 |
| Zhao et al., 2019^25^ | YES | YES | YES | YES | NO | NO | YES | YES | 6/8 |

### Opinion papers

Quality appraisal for opinion papers (n=14).

Answers: Yes, No, Unclear or Not/Applicable.

Every “Yes” or “Not Applicable (N/A)” gives a score. 0-4 scores=low quality. 5-8 scores=intermediate quality, and 9-12 scores=high quality.

| Study | Is the source of the opinion clearly identified? | Does the source of opinion have standing in the field of expertise? | Are the interests of the relevant population the central focus of the opinion? | Is the stated position the result of an analytical process, and is there logic in the opinion expressed? | Is there reference to the extant literature? | Is any incongruence with the literature/sources logically defended? | Rating |
| --- | --- | --- | --- | --- | --- | --- | --- |
| Ahmed et al., 2022^26^ | YES | YES | YES | YES | YES | N/A | 6/6 |
| Alhaffar et al., 2023^27^ | YES | YES | YES | YES | YES | N/A | 6/6 |
| Al-Mekhlafi, 2018^28^ | YES | YES | YES | YES | YES | YES | 6/6 |
| Al-Moujahed et al., 2017^29^ | YES | YES | YES | YES | YES | N/A | 6/6 |
| Al-Tammemi et al., 2022^30^ | YES | YES | YES | YES | YES | N/A | 6/6 |
| Center for Disease Control and Prevention (CDC) 2003^31^ | YES | YES | YES | YES | YES | N/A | 6/6 |
| Chumachenko and Chumachenko, 2022^32^ | YES | YES | YES | YES | YES | N/A | 6/6 |
| Dahl et al., 2022^33^ | YES | YES | YES | YES | YES | N/A | 6/6 |
| Elhadi and Msherghi, 2020^34^ | YES | YES | YES | YES | YES | N/A | 6/6 |
| Gostin et al., 2019^35^ | YES | YES | YES | YES | YES | N/A | 6/6 |
| Ilunga Kalenga  et al., 2019^36^ | YES | YES | YES | YES | YES | N/A | 6/6 |
| Martins et al., 2009^37^ | YES | YES | YES | YES | YES | YES | 6/6 |
| Mobula et al., 2020^9^ | YES | YES | YES | YES | YES | N/A | 6/6 |
| Mohamed et al., 2022^38^ | YES | YES | YES | YES | YES | YES | 6/6 |
| Nakkazi 2018^39^ | YES | YES | YES | YES | YES | N/A | 6/6 |
| Pembi et al., 2020^40^ | YES | YES | YES | YES | YES | YES | 6/6 |
| Qadri et al., 2017^41^ | YES | YES | YES | YES | YES | N/A | 6/6 |
| Quinn et al.,  2021^42^ | YES | YES | YES | YES | YES | N/A | 6/6 |
| Uwishema et al., 2022^43^ | YES | YES | YES | YES | YES | N/A | 6/6 |

### Quasi-experimental studies

Quality assessment of the included quasi-experimental studies (n=1).

Answers: Yes, No, Unclear or Not/Applicable.

Every “Yes” or “Not Applicable (N/A)” gives a score. 0-4 scores=low quality. 5-8 scores=intermediate quality, and 9-12 scores=high quality.

| Study | Is it clear in the study what is the ‘cause’ and what is the ‘effect’ (i.e. there is no confusion about which variable comes first)? | Were the participants included in any comparisons similar? | Were the participants included in any comparisons receiving similar treatment/care, other than the exposure or intervention of interest? | Was there a control group? | Were there multiple measurements of the outcome both pre and post the intervention/exposure? | Was follow up complete and if not, were differences between groups in terms of their follow up adequately described and analysed? | Were the outcomes of participants included in any comparisons measured in the same way? | Were outcomes measured in a reliable way? | Was appropriate statistical analysis used? | Rating |
| --- | --- | --- | --- | --- | --- | --- | --- | --- | --- | --- |
| Cetorelli 2015^44^ | YES | YES | YES | YES | YES | N/A | YES | YES | YES | 9/9 |

###

### Case series

Quality appraisal of the included case series studies (n=3).

Answers: Yes, No, Unclear or Not/Applicable.

Every “Yes” or “Not Applicable (N/A)” gives a score. 0-4 scores=low quality. 5-8 scores=intermediate quality, and 9-12 scores=high quality.

| Study | Were there clear criteria for inclusion in the case series? | Was the condition measured in a standard, reliable way for all participants included in the case series? | Were valid methods used for identification of the condition for all participants included in the case series? | Did the case series have consecutive inclusion of participants? | Did the case series have complete inclusion of participants? | Was there clear reporting of the demographics of the participants in the study? | Was there clear reporting of clinical information of the participants? | Were the outcomes or follow up results of cases clearly reported? | Was there clear reporting of the presenting site(s)/clinic(s) demographic information? | Was statistical analysis appropriate? | Rating |
| --- | --- | --- | --- | --- | --- | --- | --- | --- | --- | --- | --- |
| Guthmann et al., 2023^45^ | YES | YES | YES | YES | YES | YES | YES | YES | YES | N/A | 10/10 |
| Jones et al., 2020^46^ | YES | YES | YES | YES | YES | NO | YES | YES | YES | YES | 9/10 |
| Youssef et al., 2019^47^ | YES | YES | YES | YES | YES | YES | N/A | N/A | YES | N/A | 10/10 |

###

### Cohort studies

Quality appraisal of the included cohort studies (n=2).

Answers: Yes, No, Unclear or Not/Applicable.

Every “Yes” or “Not Applicable (N/A)” gives a score. 0-4 scores=low quality. 5-8 scores=intermediate quality, and 9-12 scores=high quality.

| Study | Were the two groups similar and recruited from the same population? | Were the exposures measured similarly to assign people to both exposed and unexposed groups? | Was the exposure measured in a valid and reliable way? | Were confounding factors identified? | Were strategies to deal with confounding factors stated? | Were the groups/participants free of the outcome at the start of the study (or at the moment of exposure)? | Were the outcomes measured in a valid and reliable way? | Was the follow up time reported and sufficient to be long enough for outcomes to occur? | Was follow up complete, and if not, were the reasons to loss to follow up described and explored? | Were strategies to address incomplete follow up utilized? | Was appropriate statistical analysis used? | Rating |
| --- | --- | --- | --- | --- | --- | --- | --- | --- | --- | --- | --- | --- |
| Al-Samhari et al., 2023^48^ | YES | YES | YES | YES | YES | N/A | YES | YES | YES | N/A | YES | 11/11 |
| Daw et al., 2020^49^ | NO | NO | YES | ΝΟ | NO | UNCLEAR | YES | YES | NO | N/A | Ν/Α | 5/11 |
| Katamba et al., 2020^50^ | YES | YES | YES | ΝΟ | NO | YES | YES | YES | YES | N/A | YES | 9/11 |

###

### Case report

Quality appraisal results from the case report study (n=1).

Answers: Yes, No, Unclear or Not/Applicable.

Every “Yes” or “Not Applicable (N/A)” gives a score. 0-4 scores=low quality. 5-8 scores=intermediate quality, and 9-12 scores=high quality.

| Study | Were patient’s demographic characteristics clearly described? | Was the patient’s history clearly described and presented as a timeline? | Was the current clinical condition of the patient on presentation clearly described? | Were diagnostic tests or assessment methods and the results clearly described? | Was the intervention(s) or treatment procedure(s) clearly described? | Was the post-intervention clinical condition clearly described? | Were adverse events (harms) or unanticipated events identified and described? | Does the case report provide takeaway lessons? | Rating |
| --- | --- | --- | --- | --- | --- | --- | --- | --- | --- |
| Babakura et al., 2021^51^ | YES | YES | YES | YES | YES | NO | YES | YES | 7/8 |

### Prevalence studies

Quality appraisal results from the prevalence study (n=1).

Answers: Yes, No, Unclear or Not/Applicable.

Every “Yes” or “Not Applicable (N/A)” gives a score. 0-4 scores=low quality. 5-8 scores=intermediate quality, and 9-12 scores=high quality.

| Study | Was the sample frame appropriate to address the target population? | Were study participants sampled in an appropriate way? | Was the sample size adequate? | Were the study subjects and the setting described in detail? | Was the data analysis conducted with sufficient coverage of the identified sample? | Were valid methods used for the identification of the condition? | Was the condition measured in a standard, reliable way for all participants? | Was there appropriate statistical analysis? | Was the response rate adequate, and if not, was the low response rate managed appropriately? | Rating |
| --- | --- | --- | --- | --- | --- | --- | --- | --- | --- | --- |
| Lam et al., 2017^52^ | YES | YES | YES | YES | YES | NO | NO | YES | YES | 7/9 |

# References

1. Page MJ, McKenzie JE, Bossuyt PM, Boutron I, Hoffmann TC, Mulrow CD, et al. The PRISMA 2020 statement: an updated guideline for reporting systematic reviews. BMJ 2021;372:n71. doi: 10.1136/bmj.n71
2. Moola S, Munn Z, Tufanaru C, Aromataris E, Sears K, Sfetic R, et al. Chapter 7: Systematic reviews of etiology and risk. 2019.
3. Vasylyeva TI, Liulchuk M, Friedman SR, Sazonova I, Faria NR, Katzourakis A, et al. Molecular epidemiology reveals the role of war in the spread of HIV in Ukraine. Proceedings of the National Academy of Sciences. 2018;115(5):1051-6.
4. Sedda L, Qi Q, Tatem AJ. A geostatistical analysis of the association between armed conflicts and Plasmodium falciparum malaria in Africa, 1997–2010. Malaria Journal. 2015;14(1):500.
5. Simpson RB, Babool S, Tarnas MC, Kaminski PM, Hartwick MA, Naumova EN. Signatures of Cholera Outbreak during the Yemeni Civil War, 2016&ndash;2019. International Journal of Environmental Research and Public Health. 2022;19(1):378.
6. Wells CR, Pandey A, Ndeffo Mbah ML, Gaüzère B-A, Malvy D, Singer BH, et al. The exacerbation of Ebola outbreaks by conflict in the Democratic Republic of the Congo. Proceedings of the National Academy of Sciences. 2019;116(48):24366-72.
7. Tarnas MC, Desai AN, Lassmann B, Abbara A. Increase in vector-borne disease reporting affecting humans and animals in Syria and neighboring countries after the onset of conflict: A ProMED analysis 2003-2018. Int J Infect Dis. 2021;102:103-9.
8. Haddison E, Julius C, Kagina B. Health services utilisation before and during an armed conflict; Experiences from the Southwest region of Cameroon. 2020.
9. Mobula LM, Samaha H, Yao M, Gueye AS, Diallo B, Umutoni C, et al. Recommendations for the COVID-19 Response at the National Level Based on Lessons Learned from the Ebola Virus Disease Outbreak in the Democratic Republic of the Congo. Am J Trop Med Hyg. 2020;103(1):12-7.
10. Akil L, Ahmad HA. The recent outbreaks and reemergence of poliovirus in war and conflict-affected areas. International Journal of Infectious Diseases. 2016;49:40-6.
11. Alawieh A, Musharrafieh U, Jaber A, Berry A, Ghosn N, Bizri AR. Revisiting leishmaniasis in the time of war: the Syrian conflict and the Lebanese outbreak. International Journal of Infectious Diseases. 2014;29:115-9.
12. Alghazali K, Teoh B-T, Loong S-K, Sam S-S, Che-Mat-Seri N-A-A, Samsudin N-I, et al. Dengue Outbreak during Ongoing Civil War, Taiz, Yemen. Emerging Infectious Disease journal. 2019;25(7):1397.
13. Ali A, Nisar M, Idrees M, Ahmad H, Hussain A, Rafique S, et al. Prevalence of HBV infection in suspected population of conflict-affected area of war against terrorism in North Waziristan FATA Pakistan. Infection, Genetics and Evolution. 2012;12(8):1865-9.
14. Altmann M, Suarez-Bustamante M, Soulier C, Lesavre C, Antoine C. First Wave of the 2016-17 Cholera Outbreak in Hodeidah City, Yemen - ACF Experience and Lessons Learned. PLoS Currents. 2017;9.
15. Betsi NA, Koudou BG, Cissé G, Tschannen AB, Pignol AM, Ouattara Y, et al. Effect of an armed conflict on human resources and health systems in Côte d'Ivoire: Prevention of and care for people with HIV/AIDS. AIDS Care. 2006;18(4):356-65.
16. Sobe Jermano Boyong C, Kankya C, James M, Munyeme M, Jubara AS, Ndoboli D, et al. Dynamics of tuberculosis in Wau, South Sudan during a period of armed conflict. Journal of Clinical Tuberculosis and Other Mycobacterial Diseases. 2018;12:54-65.
17. Emergency Measles Control Activities—Darfur, Sudan, 2004. JAMA. 2004;292(22):2716-8.
18. Daw MA, El-Bouzedi AH, Ahmed MO. The Impact of Armed Conflict on the Prevalence and Transmission Dynamics of HIV Infection in Libya. Front Public Health. 2022;10:779778.
19. Dureab FA, Shibib K, Al-Yousufi R, Jahn A. Yemen: Cholera outbreak and the ongoing armed conflict. J Infect Dev Ctries. 2018;12(5):397-403.
20. Gele AA, Bjune GA. Armed conflicts have an impact on the spread of tuberculosis: the case of the Somali Regional State of Ethiopia. Conflict and Health. 2010;4(1):1.
21. Haque U, Naeem A, Wang S, Espinoza J, Holovanova I, Gutor T, et al. The human toll and humanitarian crisis of the Russia-Ukraine war: the first 162 days. BMJ Global Health. 2022;7(9):e009550.
22. Malik MA, Akhtar SN, Albsoul RA, Alshyyab MA. Conflict driven displacement and child health: Evidence based on mother’s nationality from Jordan Population and Family Health Survey. PLOS ONE. 2021;16(9):e0257080.
23. Njoh AA, Mboke E, Ndoula ST, Bachir HB, Nembot R, Chebo C, et al. COVID-19 in a region of Cameroon hit by armed conflict. Pan Afr Med J. 2022;41:32.
24. Weil LM, Williams MM, Shirin T, Lawrence M, Habib ZH, Aneke JS, et al. Investigation of a Large Diphtheria Outbreak and Cocirculation of Corynebacterium pseudodiphtheriticum Among Forcibly Displaced Myanmar Nationals, 2017–2019. The Journal of Infectious Diseases. 2021;224(2):318-25.
25. Zhao Y, Lafta R, Hagopian A, Flaxman AD. The epidemiology of 32 selected communicable diseases in Iraq, 2004-2016. Int J Infect Dis. 2019;89:102-9.
26. Ahmed SH, Nashwan AJ. Cholera outbreak amid civil war: A public health crisis in Syria. J Infect Public Health. 2022;15(12):1484-1485. doi:10.1016/j.jiph.2022.11.013
27. Alhaffar MBA, Gomez MDMM, Sigua JA, Eriksson A. The cholera outbreak in Syria: a call for urgent actions. IJID Reg. 2023;8:71-74. doi:10.1016/j.ijregi.2023.06.005
28. Al-Mekhlafi H. Yemen in a Time of Cholera: Current Situation and Challenges. The American Journal of Tropical Medicine and Hygiene. 2018;98.
29. Al-Moujahed A, Alahdab F, Abolaban H, Beletsky L. Polio in Syria: Problem still not solved. Avicenna J Med. 2017;7(2):64-6.
30. Al-Tammemi AB, Sallam M. The current cholera menace amid the war crisis in Syria and the economic crisis in Lebanon: A time for global solidarity. New Microbes New Infect. 2022;51:101069. doi:10.1016/j.nmni.2022.101069
31. Prevention CfDCa. Cholera epidemic after increased civil conflict--Monrovia, Liberia, June-September 2003. MMWR Morb Mortal Wkly Rep. 2003;52(45):1093-5.
32. Chumachenko D, Chumachenko T. Impact of war on the dynamics of COVID-19 in Ukraine. BMJ Global Health. 2022;7(4):e009173.
33. Al-Tammemi AB, Sallam M. The current cholera menace amid the war crisis in Syria and the economic crisis in Lebanon: A time for global solidarity. New Microbes New Infect. 2022;51:101069. doi:10.1016/j.nmni.2022.101069
34. Elhadi M, Msherghi A. COVID-19 and civil war in Libya: the current situation. Pathog Glob Health. 2020;114(5):230-1.
35. Gostin LO, Kavanagh MM, Cameron E. Ebola and War in the Democratic Republic of Congo: Avoiding Failure and Thinking Ahead. JAMA. 2019;321(3):243-4.
36. Ilunga Kalenga O, Moeti M, Sparrow A, Nguyen V-K, Lucey D, Ghebreyesus TA. The Ongoing Ebola Epidemic in the Democratic Republic of Congo, 2018–2019. New England Journal of Medicine. 2019;381(4):373-83.
37. Martins JS, Zwi AB, Martins N, Kelly PM. Malaria control in Timor-Leste during a period of political instability: what lessons can be learned? Conflict and Health. 2009;3(1):11.
38. Mohamed A, Akbar IE, Chaudhury S, et al. Progress Toward Poliomyelitis Eradication - Afghanistan, January 2021-September 2022. MMWR Morb Mortal Wkly Rep. 2022;71(49):1541-1546. doi:10.15585/mmwr.mm7149a1
39. Nakkazi E. DR Congo Ebola virus outbreak: responding in a conflict zone. The Lancet. 2018;392(10148):623.
40. Pembi E, John S, Dumre SP, Ahmadu BU, Vuong NL, Ebied A, et al. Impact of political conflict on tuberculosis notifications in North-east Nigeria, Adamawa State: a 7-year retrospective analysis. BMJ Open. 2020;10(9):e035263.
41. Qadri F, Islam T, Clemens JD. Cholera in Yemen — An Old Foe Rearing Its Ugly Head. New England Journal of Medicine. 2017;377(21):2005-7.
42. Quinn VJ, Dhabalia TJ, Roslycky LL, Wilson VJ, Hansen JC, Hulchiy O, et al. COVID-19 at War: The Joint Forces Operation in Ukraine. Disaster Med Public Health Prep. 2022;16(5):1753-60.
43. Uwishema O, Sujanamulk B, Abbass M, Fawaz R, Javed A, Aboudib K, et al. Russia-Ukraine conflict and COVID-19: a double burden for Ukraine’s healthcare system and a concern for global citizens. Postgraduate Medical Journal. 2022;98(1162):569.
44. Cetorelli V. The impact of the Iraq War on neonatal polio immunisation coverage: a quasi-experimental study. J Epidemiol Community Health. 2015;69(3):226-31.
45. Guthmann JP, Fraisse P, Bonnet I, Robert J. Active tuberculosis screening among the displaced population fleeing Ukraine, France, February to October 2022. Euro Surveill. 2023;28(12):2300155. doi:10.2807/1560-7917.ES.2023.28.12.2300155
46. Jones FK, Wamala JF, Rumunu J, Mawien PN, Kol MT, Wohl S, et al. Successive epidemic waves of cholera in South Sudan between 2014 and 2017: a descriptive epidemiological study. The Lancet Planetary Health. 2020;4(12):e577-e87.
47. Youssef A, Harfouch R, El Zein S, Alshehabi Z, Shaaban R, Kanj SS. Visceral and Cutaneous Leishmaniases in a City in Syria and the Effects of the Syrian Conflict. The American Journal of Tropical Medicine and Hygiene. 2019;101(1):108-12.
48. Al-Samhari GA, Al-Mushiki GM, Tamrakar R, et al. Prevalence, aetiology, vaccination coverage and spatio-temporal pattern among patients admitted with acute bacterial meningitis to the sentinel hospital surveillance network in Yemen, 2014-20, before and during the civil war. Int J Epidemiol. 2023;52(4):1175-1186. doi:10.1093/ije/dyad047
49. Daw MA, El-Bouzedi AH, Ahmed MO, Alejenef AA. The epidemiological characteristics of COVID-19 in Libya during the ongoing-armed conflict. Pan Afr Med J. 2020;37:219.
50. Katamba A, Ogwang MD, Zamar DS, Muyinda H, Oneka A, Atim S, et al. Cango Lyec (Healing the Elephant): HIV incidence in post-conflict Northern Uganda. EClinicalMedicine. 2020;23:100408.
51. Babakura B, Nomhwange T, Jean Baptiste AE, Dede O, Taiwo L, Abba S, et al. The challenges of insecurity on implementing vaccination campaign and its effect on measles elimination and control efforts: A case study of 2017/18 measles campaign in Borno state, Nigeria. Vaccine. 2021;39:C66-C75.
52. Lam E, Al-Tamimi W, Russell SP, Butt MO-uI, Blanton C, Musani AS, et al. Oral Cholera Vaccine Coverage during an Outbreak and Humanitarian Crisis, Iraq, 2015. Emerging Infectious Disease journal. 2017;23(1):38.
